# Nacre-like MXene/Polyacrylic Acid Layer-by-Layer Multilayers as Hydrogen Gas Barriers

**DOI:** 10.1021/acsami.5c03632

**Published:** 2025-05-13

**Authors:** Yang Hyun Auh, Natalie N. Neal, Kailash Arole, Nolan A. Regis, Tran Nguyen, Shuichi Ogawa, Yasutaka Tsuda, Akitaka Yoshigoe, Miladin Radovic, Micah J. Green, Hisato Yamaguchi, Jodie L. Lutkenhaus

**Affiliations:** 1 Artie McFerrin Department of Chemical Engineering, 14736 Texas A&M University, College Station, Texas 77843, United States; 2 Department of Materials Science and Engineering, 14736 Texas A&M University, College Station, Texas 77840, United States; 3 5112 Los Alamos National Laboratory, Los Alamos, New Mexico 87545, United States; 4 College of Industrial Technology, Nihon University, 1-2-1 Izumi-cho, Narashino, Chiba 275-8575, Japan; 5 Materials Sciences Research Center, 26300 Japan Atomic Energy Agency, Sayo, Hyogo 679-5148, Japan

**Keywords:** MXene, polyacrylic acid, ionization, hydrogen bonds, gas barrier, synchrotron-radiation XPS

## Abstract

MXenes are a promising class of 2D nanomaterials and are of particular interest for gas barrier applications due to their high aspect ratio. However, MXene nanosheets naturally bear a negative charge, which prevents assembly with negatively charged polymers, such as polyacrylic acid (PAA), into gas barrier coatings. Here, we present MXene- and PAA-based layer-by-layer (MXene/PAA LbL) multilayers formed by leveraging hydrogen bonding interactions. When assembled in acidic conditions, MXene/PAA LbL multilayers exhibit conformal, pinhole-free, nacre-like structures. The MXene/PAA LbL multilayers yield high blocking capability and low permeability (0.14 ± 0.01 cc·mm·m^–2^·day^–1^·MPa^–1^) for hydrogen gas which is over 9000 times lower than uncoated niobium (Nb) substrate. These nacre-like structures are also electronically conductive (σ_DC_, up to 370 ± 30 S cm^–1^). Because these multilayers utilize hydrogen bonding, their properties are highly sensitive to the pH of the assembly and its external environment. Specifically, the reversible deconstruction of these multilayers under basic conditions is experimentally verified. This study shows that hydrogen bonding interactions can be leveraged to form MXene LbL multilayers as gas barriers, electronically conductive coatings, and deconstructable thin films via pH control.

## Graphical abstract

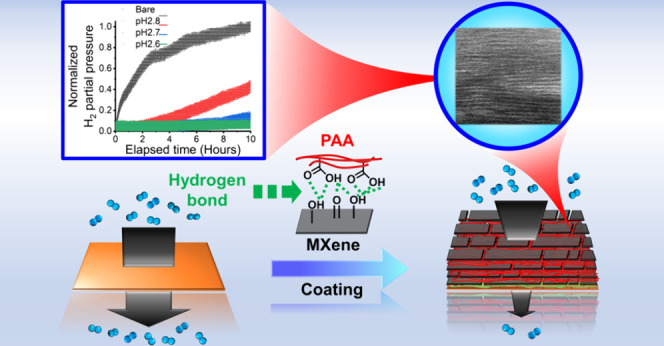

## 1 Introduction

Gas barriers, which efficiently prevent the transport of gases from one volume to another, are important for gas separations, food preservation, and infrastructure protection.^1–5^ Hybrid organic–inorganic composites are a class of promising gas barriers because the inorganic filler material (i.e., 2D nanomaterials) can alter the gas diffusion path in the organic (i.e., polymeric) matrix.^6,7^ Specifically, 2D nanomaterials can provide a tortuous diffusion path due to the nanomaterials’ high aspect ratio (*A*_R_).^1,8,9^ Besides the *A*_R_, the loading and alignment of the 2D nanomaterial in the polymer matrix strongly influence the tortuosity. Low free volume is also an important factor in slowing gas diffusion within the polymeric matrix.^10–12^ As the free volume increases, gas molecules can move more freely across the film, decreasing the gas barrier performance. Here, we hypothesized that 2D MXene nanosheets would provide effective gas diffusion barrier properties due to the MXene nanosheets’ high *A*_R_.

Self-assembled hybrid inorganic–organic brick-and-mortar, nacre-like structures are emerging as promising gas barriers.^2,13–16^ These highly ordered inorganic and polymer-based structures can form tortuous diffusion paths to prevent the transport of gas molecules. Examples of gas barrier films utilizing inorganic/organic materials include XAl-layered double hydroxide (XAl-LDH)/polyacrylic acid (PAA),^1^ mica/polylactic acid (PLA),^13^ MXene/TEMPO-oxidized nanocellulose (TNF),^2^ and f-MXene/polyvinyl alcohol^17^ have been reported. Methods of formation include layer-by-layer (LbL) assembly,^1^ blade coating,^13^ and solution casting.^17^

Titanium carbide-based MXene (Ti_3_C_2_T_*x*_ where T is a surface terminal group, such as −O, −F, or −OH) 2D nanosheets are promising candidates for nanocomposites due to the nanosheets’ large surface area, excellent mechanical properties (tensile strength of 17.3 ± 1.6 GPa and Young’s modulus of 333 ± 30 GPa),^18^ metallic electrical conductivity (σ_DC_) (∼2.4 × 10^4^ S cm^–1^),^19^ and adjustable surface functional groups.^20,21^ Thus, MXenes are currently explored across many applications, such as humidity sensors,^22^ energy storage,^23^ electromagnetic interference shielding (EMI),^24^ and gas barriers.^2,25^

PAA has been proposed as a gas barrier matrix in composite films in several past reports.^26,27^ PAA contains abundant carboxyl and carboxylate groups (COOH or COO^–^), enabling it to efficiently interact with inorganic materials.^1,28^ Being a weak polyelectrolyte with a p*K*_a_ of ∼pH 4.8,^29,30^ the relative ratio of COOH and COO^–^ groups in PAA is very sensitive to pH. As reported previously,^31^ the carboxyl groups of PAA are deprotonated at high pH, resulting in weaker hydrogen bonding interactions and increased electrostatic interactions. In contrast, at lower pH values, PAA becomes increasingly protonated, resulting in the formation of COOH groups that act as strong hydrogen bond donors. Therefore, the pH of assembly is expected to strongly influence the degree to which PAA interacts with the nanomaterial, especially if it is pH-sensitive and negatively charged like MXene nanosheets.

MXenes and PAA have been utilized as gas barrier materials separately,^1,2,17^ but the combination of the two materials is rare. In a previous study,^32^ Ti_3_C_2_T_*x*_ MXene and PAA were blended into a single composite film system using vacuum-assisted filtration. It was revealed that PAA interacted with MXenes, enhancing the swelling stability of the film, which resulted in improved nanofiltration performance for the removal of dye from water. However, PAA ionization was not specifically controlled, resulting in an unaligned and noncompact film structure. The lack of reports on MXene-PAA composites is due to the electrostatic repulsion between the two negatively charged materials, making them incompatible. MXenes bear a negative charge due to their surface terminal groups, and PAA bears a negative charge when the pH value is generally greater than 3.^33,34^

Here, we report the creation of carbide MXene (Ti_3_C_2_T_*x*_) and PAA-based LbL multilayers formed by hydrogen bonding interactions for gas barrier applications. PAA was selected because at low pH conditions (<pH 3), the ionization of the carboxyl groups in PAA is mostly inhibited, resulting in the presence of COOH instead of COO^—^.^29^ MXenes have −OH terminal groups that are capable of hydrogen bonding.^35^ Therefore, we hypothesized that protonated PAA (COOH form) would efficiently interact with MXene nanosheets via strong hydrogen bonding at low pH, forming composite multilayers with a highly aligned brick-mortar-like structure. As suggested in previous reports, inorganic and polymer-based composites with well-controlled hydrogen bonding interactions can exhibit improved ion transport efficiency,^36^ good σ_DC_,^36,37^ reinforced mechanical strength,^37,38^ and high gas barrier performance.^39^ We explore the nature of hydrogen bonding for varying pH values of the LbL assembly of PAA and the MXene, showing that assembly occurs only in acidic conditions. The LbL assembly method, which alternately deposits complementary materials, enables the fabrication of films with excellent conformality, controllable nanometer-sized thickness, high adhesion stability, and highly ordered structures for many applications.^23,40–42^ We explore the layer thickness and mass as well as the resistance to hydrogen permeation, showing that the assembly of pH tunes the nature of hydrogen bonding and results in dramatically different multilayer growth and properties. As a result, highly tunable MXene-containing coatings capable of reducing hydrogen permeability are demonstrated.

## 2 Results and Discussion

### 2.1 Characterization of MXene and LbL Assembly

Ti_3_C_2_T_*x*_ MXene nanosheets were synthesized via exfoliation of the corresponding MAX phase using the acid-etching method.^43^ The obtained MXene nanosheets were characterized for their morphology, chemical functional groups, *d*-spacing, and elemental composition using scanning electron microscopy (SEM), attenuated total reflectance Fourier transform infrared (ATR-FTIR) spectroscopy, X-ray diffraction (XRD), and X-ray photoelectron spectroscopy (XPS), respectively (Figure S1 and Table S1). The SEM image presented in Figure S1a shows well-exfoliated MXene nanosheets. The functional groups identified using ATR-FTIR spectroscopy (Figure S1b) were consistent with previous reports (OH, 3558 cm^–1^; C=O, 1655 cm^–1^).^44,45^ The absence of the MAX precursor peaks in the XRD pattern indicated successful etching and exfoliation (Figure S1c). Further, the XPS survey scan in Figure S1d and Table S1 shows that the MXene nanosheets were primarily composed of oxygen, titanium, and carbon (Ti 2p, 498.2 atom %; O 1s, 22.3%; and C 1s, 27.9%). The abundant oxygen content originates from the =O, −O, and −OH terminal groups on MXene surface, which can strongly interact with the COOH groups of the PAA polymer via hydrogen bonding. For the Ti 2p peak (Figure S1e), the oxide (TiO_2_) peaks at 458.8 eV (2p_3/2_) and 464.2 eV (2p_1/2_) were smaller than those of the other peaks, indicating minimal oxidation of the synthesized MXene. Also, the presence of Ti–F peaks (459.8 eV for 2p_3/2_ and 466.2 eV for 2p_1/2_) was minimal.

Following the confirmation of the successful MXene synthesis, the MXene nanosheets were dispersed in water and used for LbL assembly with PAA in solution, as shown in Figure 1. The pH was adjusted using hydrochloric acid and sodium hydroxide solutions. The challenge was in finding the suitable pH for both the MXene dispersion and the PAA solution to maximize hydrogen bonding interactions to form uniform and stable films. To achieve this goal, the zeta-potential (ζ-potential) of each MXene dispersion and PAA solution was investigated across various pH environments (Figures 2a, S2, and S3). Both the MXene dispersion and PAA solution exhibited an increase in ζ-potential as the pH decreased, but the MXene dispersion maintained a negative ζ-potential down to pH 2.6 (−18.2 ± 7.9 mV), as shown in Figure S2. In contrast, PAA solution (Figure S3) exhibited a shift of the ζ-potential from negative to weakly positive at pH 2.6 (0.6 ± 3.3 mV) due to the conversion of the carboxyl groups from COO^—^ to COOH, indicating that PAA could effectively form strong hydrogen bonding interactions with MXene. The colloidal stability and pH of the MXene dispersions (Figures S4 and S5) did not change significantly over 4 days, even at pH 2.6. At pH values lower than 2.6, we observed MXene oxidation and flocculation, as reported previously^33^ and shown in Figure S4.

**Figure 1:**
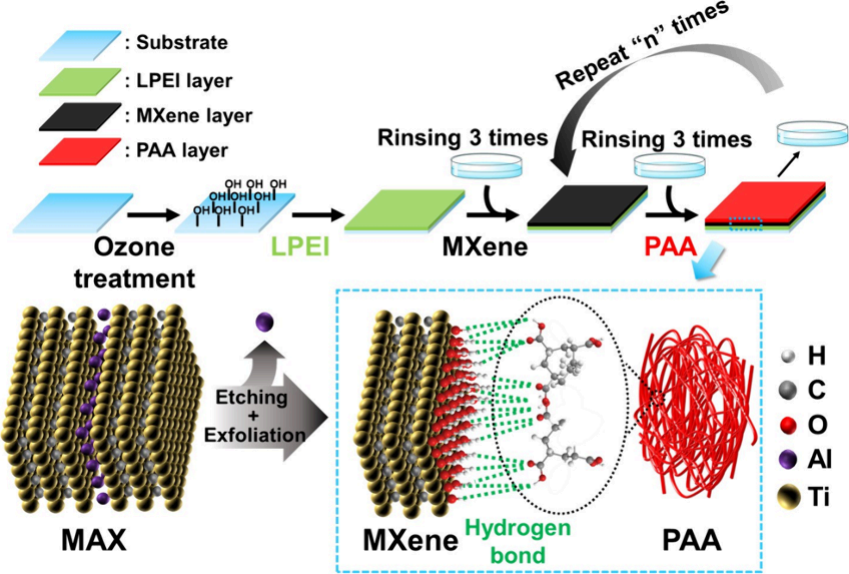
Illustration of the LbL assembly of Ti_3_C_2_T_*x*_ MXene and PAA and (bottom right) hydrogen bonding interactions between the two materials.

**Figure 2:**
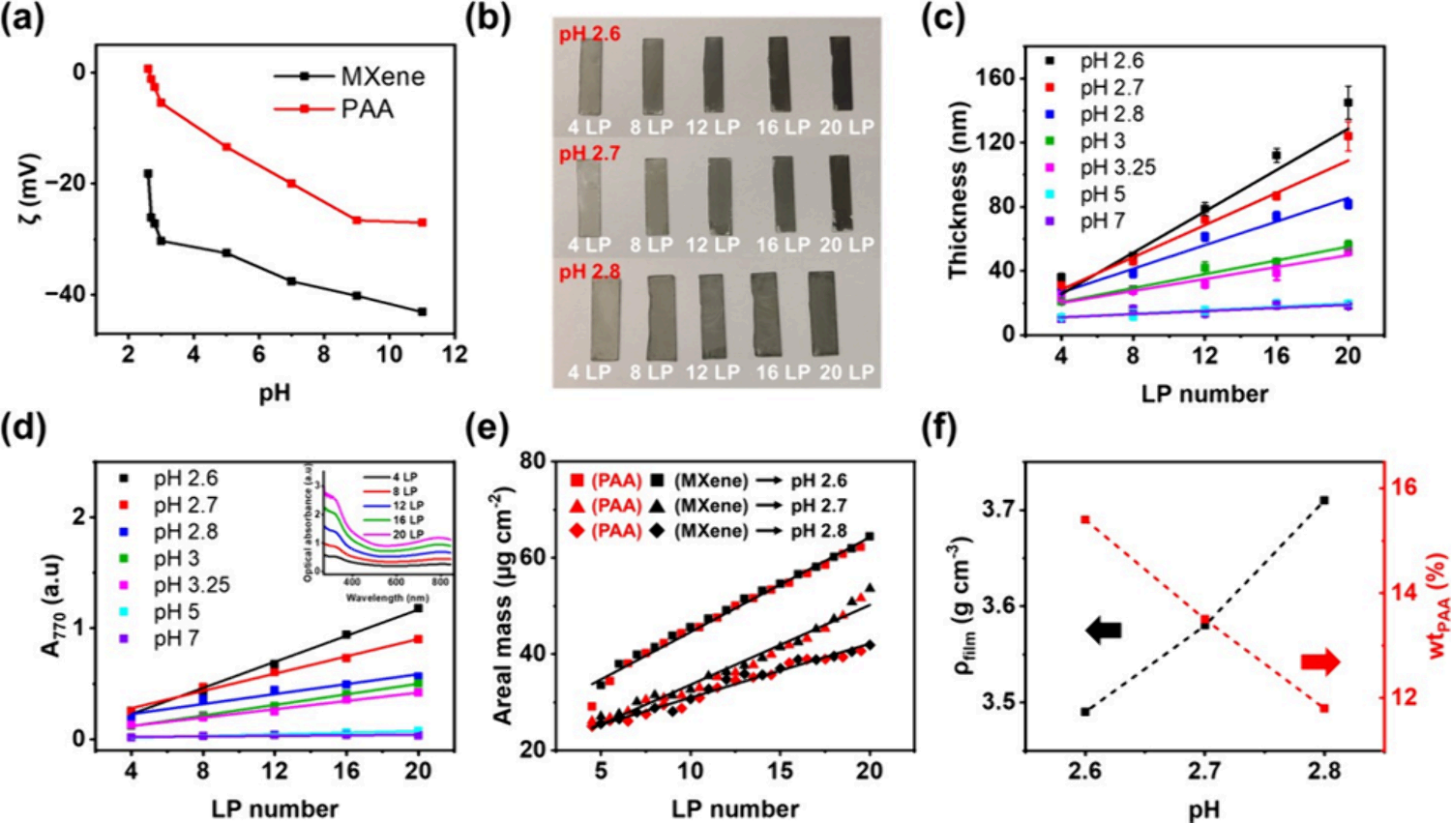
(a) ζ-potential of MXene dispersions (0.05 mg mL^–**1**^) and PAA solutions (0.05 mg mL^–**1**^) at different pH values. (b) Digital images of pH 2.6, 2.7, and 2.8 MXene/PAA LbL assemblies with varying LP numbers. (c) Profilometric thickness and (d) optical absorbance intensity at 770 nm (*A*_770_) of MXene/PAA LbL assemblies deposited at pH values from 2.6 to 7.0. The inset of (d) is the full optical absorbance spectrum of pH 2.6 LbL multilayers. (e) Areal mass of pH 2.6, 2.7, and 2.8 LbL multilayers as a function of LP number. The areal masses of MXene and PAA are plotted as filled black and red marks, respectively. The different marker shapes represent each different pH values of assembly. (f) Changes in density and PAA weight percentage (wt_PAA_) as a function of assembly pH. The LP number for density and mass calculation was fixed at 20.

Next, MXene/PAA-based composite multilayers were fabricated by using the LbL assembly method. Citric acid was added into the PAA solutions, MXene dispersions, and Milli-Q water rinses to prevent MXene oxidation.^33,46^ Similarly, each LbL assembly bath was adjusted to be the same pH. Linear polyethylenimine (LPEI) was adsorbed directly onto the substrates as an anchor layer to enhance MXene attachment during the initial stages of LbL growth.^23^ MXene and PAA were alternately assembled onto the substrate until the desired layer pair (LP) number was reached. For each multilayer, the LPEI layer was applied only once to the substrates and the last layer was MXene. Figures 2b and S6 show that the LbL multilayers became darker with increasing LP number and decreasing pH. The film thickness (Figures 2c and S10), film roughness (Figure S7), optical transmittance (Figure S8a), and optical absorbance spectra (Figures 2d and S8b–S10) were measured, and linear growth was observed in all MXene/PAA LbL multilayers with the increase of LP number. Notably, the film thickness and optical absorbance at 770 nm (*A*_770_) increased dramatically as the pH of the assembly decreased below 3.0 (Figure S10). At higher values of assembly pH, the LbL multilayer growth decreased and became minimal (see pH 5 and 7 in Figure 2c). This can be explained by the carboxyl groups within PAA converting from COO^—^ to COOH at acidic pH levels, enabling a greater number of hydrogen bonding interactions with MXene nanosheets, promoting growth, and resulting in thicker multilayers.

We next focused our study to assembly pH values of 2.6, 2.7, and 2.8 based on the higher film thickness and growth. To quantitatively determine the MXene and PAA content, the areal mass was measured using quartz crystal microbalance (QCM) (Figures 2e and S11). As the assembly pH decreased, the average LP thickness increased (Table 1). Specifically, the average LP thickness nearly doubled when the assembly pH decreased from 2.8 to 2.6, showing the extreme sensitivity of the growth with respect to the pH. Figure 2f displays the calculated PAA weight percentage (wt_PAA_, %) and film density (ρ_film_, g cm^–3^). The wt_PAA_ and ρ_film_ values were calculated using eqs S1 and S2 in the Supporting Information, respectively. As the assembly pH decreased, wt_PAA_ increased and ρ_film_ decreased (Table 1). With the decrease in pH, more PAA repeat units could engage in hydrogen bonding with MXene, resulting in PAA’s promoted adsorption and a decrease in the film’s density.

**Table 1:** Features of pH 2.6, 2.7, and 2.8 LbL Multilayers, 20 LPs

	pH 2.6	pH 2.7	pH 2.8
Thickness^a^ (nm)	145.0 ± 10.2	124.0 ± 9.2	81.7 ± 2.9
*R*_q_^b^ (nm)	13.2	10.4	8.1
*A*_770_ ^c^	1.2	0.9	0.6
*T*^d^ (%)	6.4	12.3	17.8
ρ_film_ ^e^ (g cm^–3^)	3.49	3.58	3.71
wt_PAA_ ^f^ (%)	15.4	13.5	11.8
wt_MXene_ ^g^ (%)	84.6	86.5	88.2
No. of MXene layers per LP^h^	8.4	6.7	4.2
σ_DC_ ^i^ (S cm^–1^)	67.8 ± 1.1	46.8 ± 4.9	27.6 ± 1.7
*d*^j^ (nm)	1.65	1.64	1.63

a Thickness of the multilayer coated on glass substrates.

b Roughness of each LbL multilayer measured by AFM.

c Optical absorption intensity at 770 nm region.

d Optical transmittance at 770 nm.

e Film density measured from QCM.

f Calculated PAA weight percentage from QCM.

g Calculated MXene weight percentage from QCM.

h The number of MXene layers per layer pair.

i Electrical conductivity of each LbL multilayer.

j *d*-spacing gap of interlayer.

To explore the pH-driven hydrogen bonding interactions between MXene and PAA in more detail, pH 2.6, 2.7, and 2.8 assemblies were compared using laboratory-based XPS. The component peaks of all LbL multilayers (Figure 3 and Tables S3–S5) showed a similar binding energy position to the corresponding types of peaks in MXene (Figure S1e–g) and PAA (described in refs 23, 32, 47–53 ). For the LbL multialyers, the Ti 2p elemental percentage increased and the O 1s contribution decreased when compared to MXene alone. Specifically, the multilayer created at pH 2.6 showed especially higher atomic percentages of carbon and oxygen (O 1s, 42.5%, and C 1s, 25.6%) than the other two LbL multilayers, which is attributed to the enrichment of PAA at the pH of lower assembly. Also, in Ti 2p (Figure 3b–d), the TiO_2_ peak intensity at 458.6–458.7 eV increased slightly compared to the MXene alone (Figure S1e) due to the acidic conditions during film fabrication.

**Figure 3:**
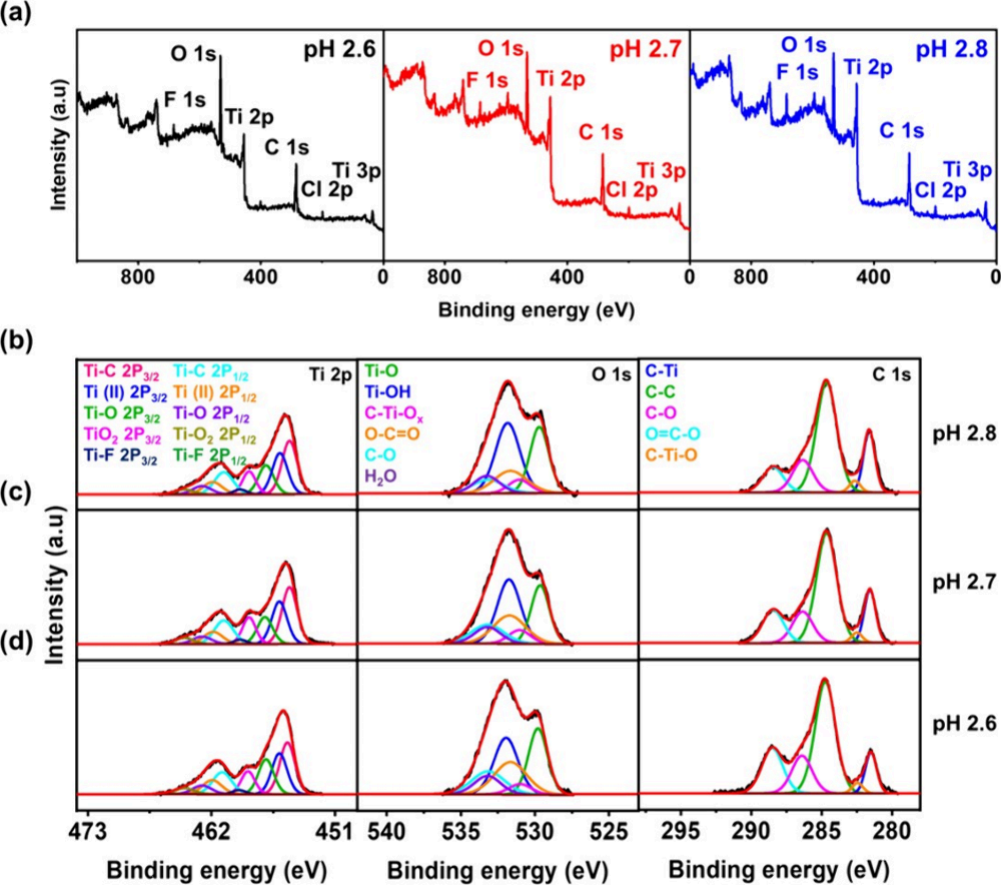
(a) XPS survey scans and component peak fittings for Ti 2p, O 1s, and C 1s of (b) pH 2.8, (c) pH 2.7, and (d) pH 2.6 MXene/PAA LbL composite multilayers measured by laboratory-based XPS.

### 2.2 Characterization of Hydrogen Bonding Interactions and Multilayer Morphology

To corroborate that the interaction between MXene and PAA inside the multilayer originates from hydrogen bonding, ATR-FTIR spectroscopy and XRD measurements were performed (Figure 4a,b). ATR-FTIR (Figure 4a) spectra showed the vibration and stretching frequencies of the functional groups present in PAA, MXene, and the pH 2.6 LbL multilayer. PAA showed stretching frequencies at 3357 cm^–1^, 1710 cm^–1^, and 1450 cm^–1^, which are respectively characteristic of OH, C=O, and COOH bonds, whereas MXene exhibited OH and C=O bonds at 3743 cm^–1^ and 1535 cm^–1^, respectively, as described in refs 44, 45, 54, 55 . The pH 2.6 LbL multilayer exhibited prominent peaks attributed to the carboxyl groups derived from both PAA and MXene. Specifically, the OH stretching frequency (3492 cm^–1^) was positioned between the corresponding peaks for PAA and MXene. On the other hand, the C=O and COOH peaks of the LbL multilayer were red-shifted to 1727 cm^–1^ and 1454 cm^–1^, respectively, compared to PAA and MXene alone. These peak shifts result from hydrogen bonding interactions between different types of materials.^56,57^ Indeed, the pH 2.6 MXene/PAA LbL multilayer exhibited the most prominent red-shifting as compared with the other multilayers assembled at higher pH values, as shown in Figure S12, which confirms the prominent interactions between MXene and PAA.

**Figure 4:**
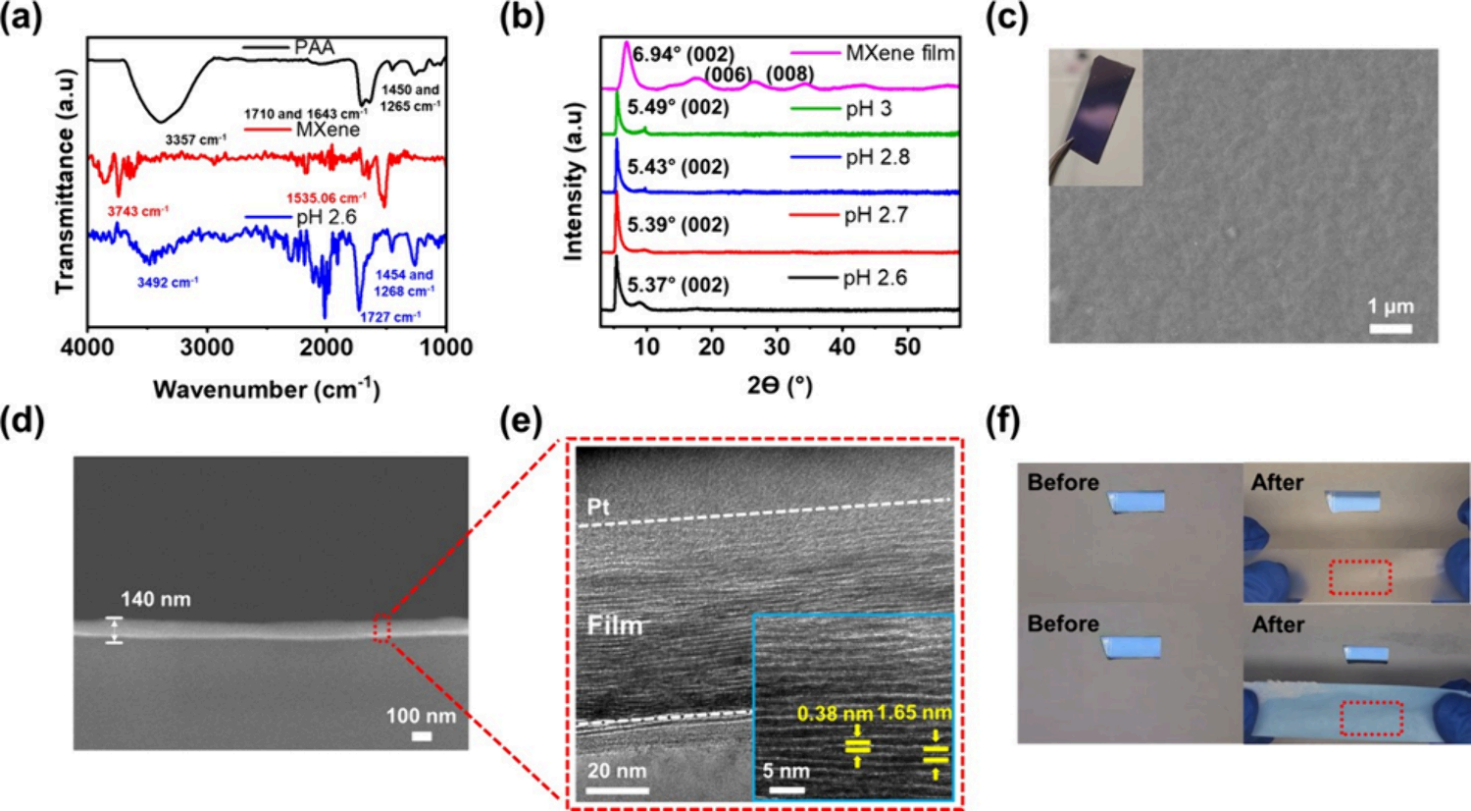
(a) FTIR spectra of PAA, a VAF MXene film, and pH 2.6 MXene/PAA LbL multilayer. (b) XRD patterns of each LbL multilayer and the VAF MXene film after baseline subtraction. (c) Digital and top-view SEM images, (d) cross-sectional SEM image, and (e) cross-sectional TEM images of pH 2.6 MXene/PAA LbL multilayers. (f) Digital images of adhesion tape test for a pH 2.6 MXene/PAA LbL multilayer using two types of tape. Transparent tape was used to check for MXene fragments, and blue tape was used to test for higher adhesion. The LP number of the multilayers for the measurements was fixed at 20.

XRD was used to probe the *d*-spacing of a MXene film made by vacuum-assisted filtration (VAF) and MXene/PAA LbL multilayers calculated using Bragg’s law^58^ and the (002) peak (Figures 4b and S13). As shown in Table 1, the pH 2.6, 2.7, and 2.8 LbL multilayers exhibited larger *d*-spacing values of 1.65, 1.64, and 1.63 nm, respectively, than the VAF MXene film (1.27 nm), indicating the successful incorporation of PAA into the interlayer of MXene. The MXene spacing of the LbL multilayers increased slightly as the pH decreased. However, despite the relatively large PAA weight percentage difference between the LbL multilayers (∼3.6%), as revealed by QCM (Figure 2e and Table 1), the difference in interlayer spacing was relatively small, ranging from 0.01 to 0.05 nm. This indicates that MXene sheets may deposit onto the substrate as few-layer flakes rather than as individual nanosheets.

SEM was used to investigate the surface and cross-sectional morphology of the MXene/PAA LbL multilayer (Figures 4c,d and S14). The LbL multilayer was assembled at pH 2.6 on a Si wafer substrate. In the top-down SEM images, the multilayer surface appeared smooth and pinhole free (Figure 4c). To further assess the surface, AFM topographic scans and phase images of MXene/PAA LbL multilayers were examined (Figure S15), confirming the uniform coverage and low surface roughness of *R*_q_ = 8–13 nm.

The cross-sectional SEM image of the LbL multilayer in Figure 4d and Figure S16 shows that the LbL multilayer also uniformly coated the Si wafer substrate. Also, the film thickness was similar to the profilometer results. The cross-sectional TEM image of the LbL multilayer (Figures 4e and S17) revealed alternating layers of MXene and PAA, forming a compact brick-and-mortar-like structure. Notably, it was found that the average thickness of the PAA region was 0.38 nm, whereas the *d*-spacing between MXene layers was measured to be 1.65 nm, aligning with the XRD results (Figure 4b). Consequently, the low roughness and highly aligned film structure of MXene/PAA LbL multilayers can be expected to contribute to the high gas barrier performance.^1,13,59^

A qualitative peel test was also implemented to demonstrate the adhesion of the MXene/PAA LbL assembly (Figures 4f and S18 and ). Two types of tape were used because transparent tape facilitates the easy observation of MXene fragments, whereas the blue tape had much stronger adhesive properties. MXene fragments were not found in any of the MXene/PAA multilayers when the tapes were applied and removed from the multilayers after a few seconds.

### 2.3 Gas Barrier Properties

Gas barrier performance is very sensitive to defects, such as pinholes and cracks. Low-density defects, which cannot necessarily be detected by other material characterization techniques due to the generally localized nature of the analysis area, may be present in our multilayers. To probe this, we performed a study of the LbL multilayer’s gas barrier properties using the configuration shown in Figure 5a. Two vacuum chambers were separated by the sample, and hydrogen gas was selected for testing because it is considered one of the most permeable gases available. Hydrogen gas was introduced from one side, and a residual gas analyzer (RGA) capable of detecting the permeating gas was placed on the opposite side. Niobium (Nb) foil was used as a gas-permeable metal substrate onto which four LPs of pH 2.6, 2.7, and 2.8 MXene/PAA multilayers were deposited. The samples were heated to 400 °C to achieve detectable hydrogen gas permeation through the Nb substrates and to render the molecular gas behavior close to that of the ideal gas state. At this temperature, PAA will decompose; thus, the results are expected to reflect mostly those of the MXene layers of the LbL composite films. This should not present a problem because the MXene nanosheet layers are expected to dominate the gas barrier properties, as described in other studies regarding 2D nanomaterial barriers.^2,60^ The gas permeability of the Nb substrate and each MXene/PAA LbL multilayer was calculated using an equation based on leak rate and saturated pressure (Table S6), as previously reported in the literature.^15,61–63^ The influence of substrate on permeability was removed by using the Henis and Tripodi resistance model.^25,64^

**Figure 5:**
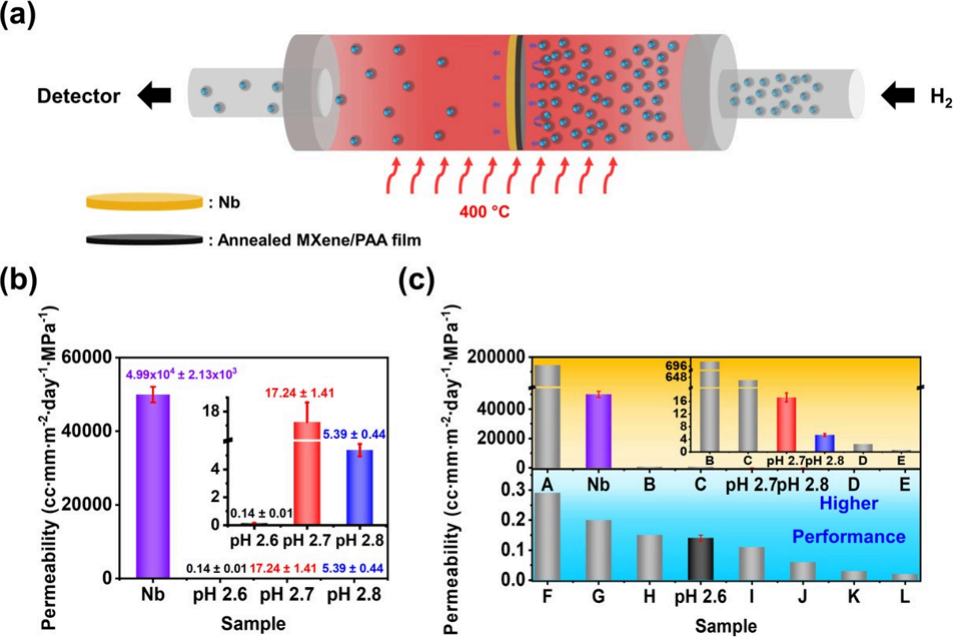
(a) Illustration scheme of gas permeability measurement under 400 °C and 20 kPa conditions. (b) H_2_ gas permeability of a bare Nb substrate and MXene/PAA LbL multilayers. The inset is the permeability of MXene/PAA LbL multilayers with enlarged *Y*-axis scale. (c) Comparison of permeabilities in our study and those reported for other composite films. The inset is the permeability of pH 2.7 and 2.8 MXene/PAA LbL multilayers and other reported composite films from B to E with enlarged *Y*-axis scale. The characteristics of the samples from the literature are further described in Table S7.

All of the LbL multilayers exhibited extremely reduced gas permeability properties compared with the bare and uncoated Nb substrate (Figure 5b**)**. This observation is remarkable because the Nb substrate is 0.127 mm thick, whereas the deposited LbL multilayers are only from 26 to 36 nm thick, depending on assembly pH. Notably, the pH 2.6 multilayer exhibited the best gas barrier qualities, with a saturated pressure value (3.68 × 10^–7^ Pa) close to the detection limit of our system (Figure S19a, Table S6) due to the highly ordered MXene structure. The pH 2.6 LbL multilayer exhibited the lowest calculated permeance (4.03 × 10^3^ cc·m^–2^·day^–1^·MPa^–1^) and permeability (0.14 ± 0.01 cc·mm·m^–2^·day^–1^·MPa^–1^) values. Even though its annealed XRD *d*-spacing gap, shown in Figure S19b, was a value between that of the three types of MXene/PAA LbL multilayers, we expect the pH 2.6 assembly condition to bear the best barrier properties because the number of MXene layers deposited per exposure (8.4 MXene sheets, Table 1) was the highest. Therefore, the intricate nanobrick wall structure from the pH 2.6 LbL multilayer, as shown in the TEM image (Figure 4e), forms a tortuous and long diffusion pathway that efficiently prevents hydrogen gas from permeating.

In Figure 5c, the hydrogen gas permeability of the MXene/PAA LbL multilayers is compared with that of other conventional composite multilayers. The thickness and permeability information on all films are presented in Table S7. As a result, the pH 2.6 LbL multilayer exhibited comparably low gas permeability despite having the lowest thickness compared to other conventional composite films, confirming the LbL multilayer’s potential as a highly efficient gas barrier for commercial applications. Specifically, the pH 2.6 multilayer was better than coatings based on graphene oxide (GO)/polyethylenimine (PEI)^14^ but worse than those based on positively charged GO boron nitride (BN)/epoxy semi-interpenetrating networks (S-IPN)^65^ and polystyrene sulfonate (PSS)-RGO/PEI-RGO.^66^

### 2.4 Aging Effects

To understand the effects of aging, we monitored the σ_DC_ values of the LbL multilayers over time after storage in air. The σ_DC_ was calculated using eq S3 in Supporting Information, as described in ref 67. The initial electrical σ_DC_ of pH 2.6, 2.7, and 2.8 multilayers calculated from the sheet resistance was 67.8 ± 1.1, 46.8 ± 4.9, and 27.6 ± 1.7 S cm^–1^, respectively. This confirms that the σ_DC_ increased with decreasing pH of the assembly (Figure 6a). This is attributed to the difference in the number of MXene layers per LP (Table 1) in the multilayer. The pH 2.6 film showed a larger number of MXene layers per LP (number of MXene layers per LP, ∼8.4) than the other two composite multilayers (6.7 and 4.2 for pH 2.7 and 2.8, respectively, calculated using eq S4). This result implies that the quantity of MXene nanosheets regulated by varying the pH is a dominant factor in determining the σ_DC_ of the multilayers. After 120 days of storage in ambient conditions, the σ_DC_ the LbL multilayers decreased by 44.5–69.2%, indirectly indicating that some of the MXene had oxidized (Figure 6b). For practical application, oxidation may be slowed with antioxidant additives.

**Figure 6:**
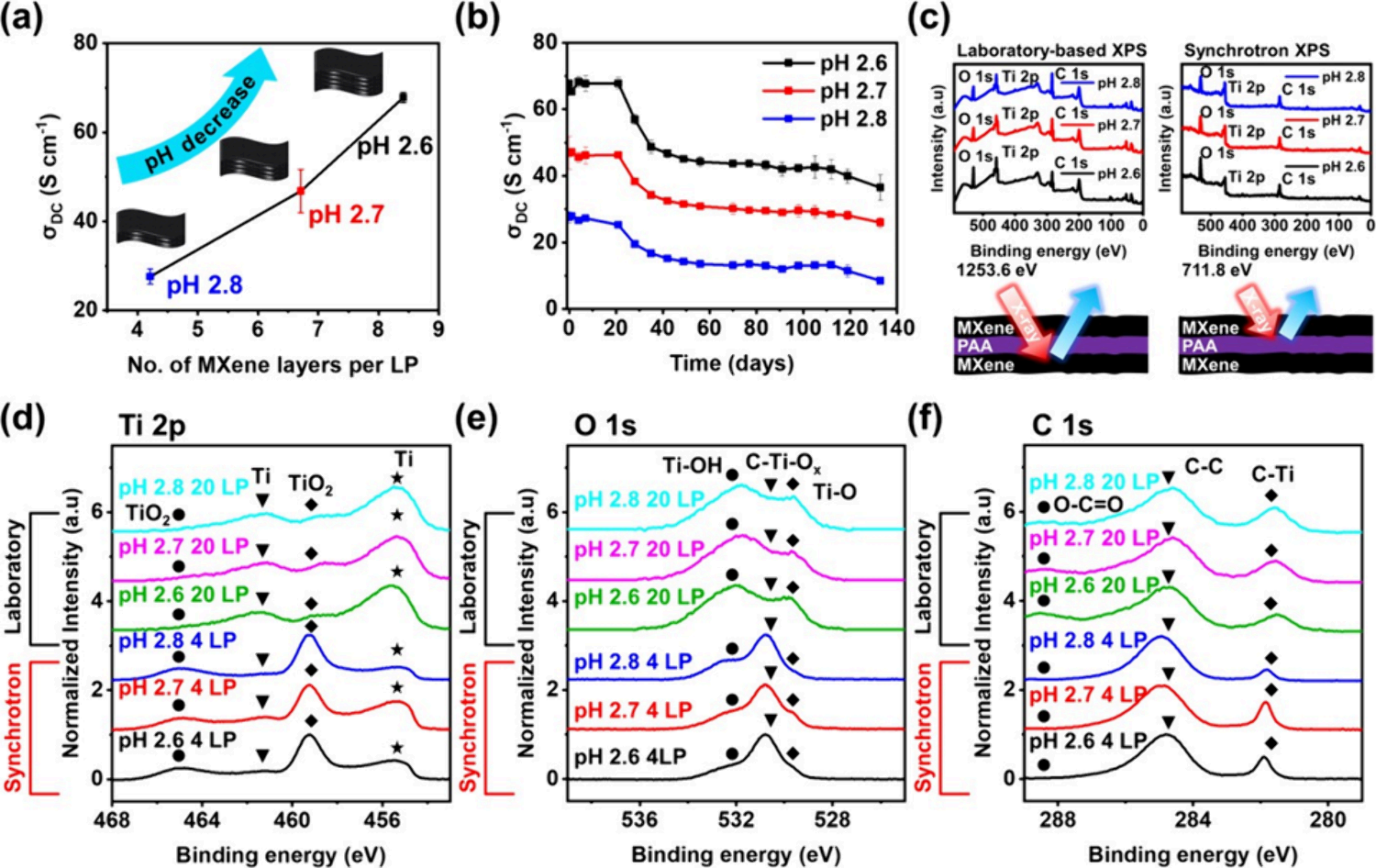
(a) σ_DC_ of LbL multilayers as a function of the number of MXene layers per LP; see Table 1. (b) σ_DC_ evolution of LbL multilayers over time. The multilayer LP number was 20. (c) Schematic illustration of analysis depth for laboratory-based and SR-XPS (top) and their survey scans (bottom). Component peaks of (d) Ti 2p, (e) O 1s, and (f) C 1s at pH 2.6, 2.7, and 2.8 based LbL multilayers.

To examine the effects of varying relative humidity (RH), the electrical conductivity and the ellipsometric film thickness of the LbL multilayers were investigated (Figure S20). The LbL multilayers showed a decrease in electrical conductivity with time for RH values of >40%. For a RH value of 20%, the conductivity was more stable. In response to increasing the RH value, the multilayer thickness first increased linearly (up to RH = 60%) and the superlinearly (above RH = 60%) due to the adsorption of water molecules into film interlayer spaces.^22^ This process increases the tunneling resistance between MXene nanosheets, causing a drop in the electrical conductivity.^22^ After redrying under RH conditions of air, the electrical conductivity of the MXene/PAA LbL multilayers recovered up to 95% due to the removal of water molecules (Figure S21).

To monitor the possible oxidation of MXene during aging, synchrotron radiation XPS (SR-XPS) was employed (Figure 6c–f and Tables S8–S10). The lower source energy for SR-XPS vs the laboratory-based XPS (i.e., 711.6 eV vs 1253.6 eV) leads to a shorter electron mean free path, allowing us to probe the surface more sensitively.^68,69^ In the results of the survey scan, the Ti 2p atomic ratio from SR-XPS decreased from 54.4% to 43.0% with increasing assembly pH (Figure 6c and Tables S8–S10), contrary to laboratory-based XPS results (Tables S3–S5). This is attributed to the decrease in the number of MXene layers per LP with an increasing assembly pH, resulting in the increased amount of PAA probed by SR-XPS beneath the MXenes on the surface. Furthermore, the TiO_2_ peaks (Figures 6d and S22a–c) were higher than those of laboratory-based XPS results, indicating oxidation at the multilayer’s outermost surface.^23,47,70^ This indicates that the oxidation of the MXene sheets in the multilayer is localized at the surface and not the interior.

### 2.5 pH Effects on Multilayer Stability

PAA ionization is anticipated under basic conditions, which we expect could trigger the on-demand deconstruction of the multilayer. To investigate this concept, as-made MXene/PAA LbL multilayers assembled at pH 2.6, 2.7, and 2.8 were immersed in pH-adjusted aqueous solutions, Figure 7a and Figures S23 and S24). In particular, for immersion in pH 11 and 13, the multilayers delaminated in less than 1 day. To examine these changes in detail, film thickness, UV absorption, and σ_DC_ were measured (Figures 7b–d and S25–S29). In Figures 7b and S25, the film thickness changed minimally in the pH 3 environment, whereas the thickness decreased sharply above pH 5. This is attributed to the ionization of PAA and the replacement of hydrogen bonding interactions between MXene and PAA with electrostatic repulsion.

**Figure 7:**
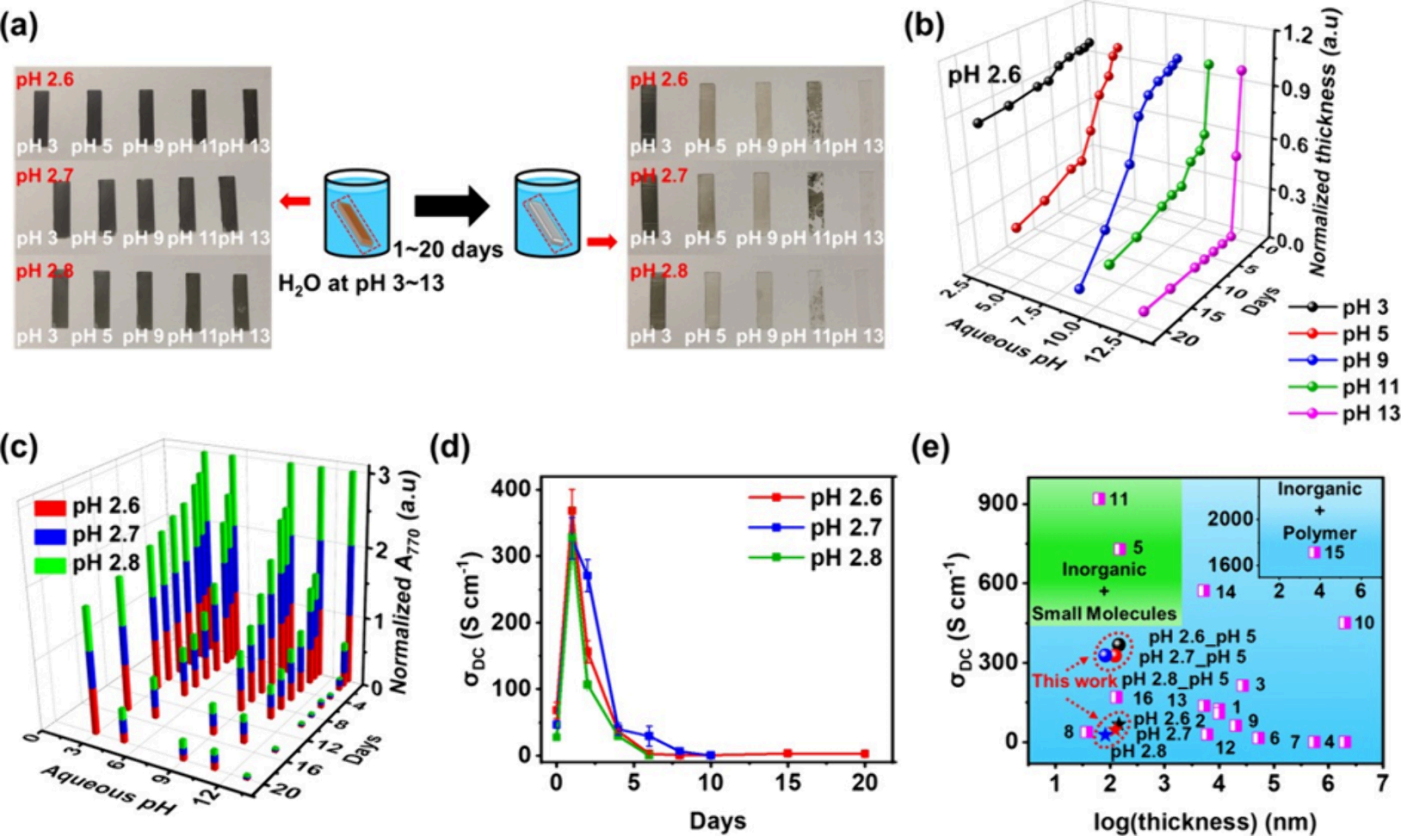
(a) Digital images and illustration scheme of each pH LbL multilayer having 20 LP before and after dipping into a pH-adjusted aqueous solution. The film pH is in red letters, and the aqueous solution pH is in white letters. The changes in (b) normalized film thickness of pH 2.6 multilayer and (c) optical absorbance at 770 nm as a function of aqueous pH and time. The colored lines and markers indicate different aqueous pH values. (d) Time-dependent σ_DC_ changes of pH 2.6, 2.7, and 2.8 LbL multilayers after immersion in pH 5 aqueous solution. (e) Comparison of electrical conductivity for LbL multilayers fabricated in this work and others reported in the literature. The pH 2.6, 2.7, and 2.8_pH 5 indicate the pH 2.6, 2.7, and 2.8 MXene/PAA LbL multilayers after immersion in pH 5 aqueous solution for 1 day, respectively.

UV–vis spectroscopy (Figure 7c) of the multilayers after immersion in pH 5 generally showed a decrease in the absorption intensity of the MXene region (at 770 nm), confirming multilayer deconstruction. The pH 2.6 multilayer exhibited the greatest stability compared to other LbL multilayers, as the film thickness and the normalized *A*_770_ decreased more slowly (Figures 7b,c and S25–S28). This may be due to the more numerous hydrogen bonding interaction in the original multilayer assembled at pH 2.6 multilayer.

The σ_DC_ of the pH 2.6, 2.7, and 2.8 multilayers was also measured after immersing in pH 5 aqueous solution (Figures 7d and S29). Interestingly, the σ_DC_ of pH 2.6, 2.7, and 2.8 multilayers increased dramatically from 68 ± 1, 47 ± 5, and 28 ± 2 S cm^–1^ to 370 ± 30, 330 ± 30, and 330 ± 50 S cm^–1^, respectively, after immersion in pH 5 aqueous solution for 1 day. The increased σ_DC_ values are superior to or comparable to those of most other conventional inorganic and polymer-based composite films, as shown in Figure 7e and Table S11. This indicates that brief immersion might be used to enhance electrical conductivity.

## 3 Conclusions

MXene and PAA-based LbL assemblies were successfully created, and the pH-dependent tunability of the film properties was explored for gas barrier applications. The assembly of these materials could only be achieved at low pH values, where PAA is protonated, and hydrogen bonding interactions between PAA and MXene are prominent. Specifically, the pH 2.6 LbL multilayer exhibited the largest number of MXene-PAA hydrogen bonding interactions, as confirmed using FTIR spectroscopy. Further, the pH 2.6 LbL multilayer displayed the highest PAA content but the largest number of MXene layers deposited per exposure. As confirmed by SEM, AFM, and TEM, the MXene/PAA multilayers showed a uniform surface, low roughness, and compact brick-mortar-like structure. The MXene/PAA LbL multilayers exhibited good adhesion during tape testing. The gas barrier performance of the pH 2.6 MXene/PAA LbL multilayer at high temperature (400 °C) exhibited the lowest permeability (over 9000 times lower than that of its Niobium substrate), which we attributed to the tortuous pathways provided by the larger number of MXene sheets deposited per LP. For the same reason, the pH 2.6 MXene/PAA LbL assembly also exhibited the highest σ_DC_. Leveraging the reversibility of hydrogen bonding interactions, we demonstrated that MXene/PAA LbL multilayers deconstructed more rapidly in basic environments than in acidic environments due to the ionization of PAA. Overall, this work demonstrated that hydrogen bonding interactions are important for incorporating MXenes into highly tunable LbL multilayers for gas barrier applications.

## 4 Experimental Section

### 4.1 Materials

Lithium fluoride (LiF), dimethyl sulfoxide (DMSO), polyacrylic acid (PAA, M_W_ = 100 000 g mol^–1^, 35 wt % in water), hydrochloric acid (HCl, ACS reagent, 37% w/w), and sodium hydroxide (NaOH) were purchased from Sigma-Aldrich. Citric acid, isopropyl alcohol (99%), and ethanol (99.5%) were purchased from BDH. Linear polyethylenimine (LPEI, M_W_ = 100 000 g mol^–1^) was purchased from Polysciences Inc. The purification of water was carried out to obtain 18.2 MΩ (Milli-Q) water. Slide glasses (75 mm × 25 mm × 1 mm) and Si wafers, which were used for LbL deposition, were purchased from VWR and University Wafer, respectively. Ti/Au quartz crystal substrates (5 MHz) for QCM measurements were purchased from Inficon. Niobium (0.127 mm, 0.005in) thick, 99.97% (metals basis excluding Ta, Ta |<0.06%) substrates for testing gas permeation were purchased from ThermoFisher.

### 4.2 Synthesis of Ti_3_C_2_T_*x*_ MXene

Initially, 0.8 g of lithium fluoride was gradually added into 6 M hydrochloric acid (10 mL) while maintaining constant magnetic stirring at 40 °C. Subsequently, 1 g of Ti_3_AlC_2_ powder was slowly added to the LiF and HCl mixture at 40 °C for 40 h with continuous magnetic stirring. In this step, MAX phase powder should be added very slowly to prevent exothermic reactions. Following the 40 h period, the solution underwent transfer and centrifugation at 9000 rpm for 15 min to eliminate hydrofluoric acid (HF). The resulting sediment was redispersed in water and underwent three to four washing cycles to achieve a neutral pH. Subsequently, the sediment underwent resuspension in DMSO for intercalation and was stirred for 20 h. Sequential water washes (2–3 times) were conducted, and finally, the sediment underwent bath sonication in water for 1 h. The supernatant consisting of delaminated MXenes was collected after the last centrifugation at 3500 rpm for 55 min and subsequently freeze-dried for approximately 24 h to obtain Ti_3_C_2_T_*x*_ nanosheets.

### 4.3 Preparation of MXene/PAA Composite Multilayer

The PAA and MXene nanosheets were, respectively, dissolved/suspended into Milli-Q water at a concentration of 1.0 mg mL^–1^ with the addition of citric acid (1.0 mg mL^–1^). The pH of all solutions was fixed at the same value. A substrate (glass or silicon wafer) was cut into 12.5 mm × 50.0 mm or 12.5 mm × 25.0 mm. The cut substrates were dipped into isopropyl alcohol and underwent sonication for 15 min. The sonication treated substrates were rinsed with Milli-Q water and acetone and then dried with high velocity air. After washing, the substrates were plasma-treated (Harrick PDC-32G) for 15 min. Then, the substrates were immersed in pH-adjusted LPEI aqueous solution (1 mg mL^–1^) containing citric acid (1 mg mL^–1^) for 15 min and rinsed three times for 10 s each using pH-adjusted Milli-Q water containing 1 mg mL^–1^ of citric acid. The LPEI-coated samples were then immersed in MXene dispersion for 15 min and rinsed three times for 10 s each with aqueous solution. PAA was assembled on the sample in the same way as LPEI and MXene until the desired thickness of the sample was obtained by repeating this procedure. The last layer of all of the samples consisted of MXene.

### 4.4 Characterization

Synthesized MXene materials were characterized using scanning electron microscopy (SEM, JEOL JSM-7500F), X-ray diffraction (XRD, Bruker D8 X-ray diffractometer), ATR-FTIR spectroscopy (Nicolet iS5 spectrophotometer (Thermo Scientific) equipped with an attenuated total reflectance (ATR, iD7)), and X-ray photoelectron spectroscopy (XPS, Omicron XPS/UPS system with Argus detector) after freeze-drying. The laboratory-based XPS measurement conditions were fixed as the same for MXene and all LbL multilayers. The XPS source energy was fixed at 1253.6 eV. CasaXPS software was used to deconvolute the component spectra following previous reports.^23,47–51^ A Shirley type background function was employed to establish background contributions in all spectra, while Gaussian–Lorentzian curves were utilized to fit all peaks. Peak fitting for MXenes involved several major constraints: component binding energies were limited to within ±0.4 eV of the initial literature values.^23,47–49^ Full-width half-maximum (fwhm) values were also restricted to similar chemical bonds or atoms to have values similar to each other. Additionally, constraints were applied to ensure an area ratio of 2p_3/2_ and 2p_1/2_ of around 2:1 for all components in the Ti 2p spectra at first. Furthermore, asymmetric peaks were used for fitting C–Ti–T_*x*_ peaks due to their conducting nature. Elements were split into distinct components. The pH of every solution used in this work was adjusted by a pH meter (Accument AB 15 Basic). The ζ-potential of Ti_3_C_2_T_*x*_ MXene dispersion and PAA aqueous solution were measured using a Zeta-sizer (Malvern Zetasizer NanoZS). LbL multilayers on glass substrates were used to measure profilometric film thickness, profilometry roughness, UV–vis absorbance and transmittance, ATR-FTIR spectra, XRD, electrical conductivity, pH stability experiments, and ellipsometric film thickness. LbL multilayers on Si substrates were used to measure laboratory-based XPS, SEM, TEM, tape test, and synchrotron-radiation XPS. The film thickness and roughness (*R*_q_) were recorded using an Alpha step profilometer (KLA Tencor D-100). The UV/vis absorption and transmittance measurements were accomplished using a UV/vis spectrometer (Shimadzu SolidSpec-3700 UV–vis–NIR). The mass profile, component weight percentage, film density, and the number of MXene layers per layer pair of MXene/PAA LbL multilayers were obtained using a quartz crystal microbalance (QCM, MAXTEKRQCM Research) and each LbL multilayer was coated on 5 MHz Ti/Au quartz crystal sensors. Frequency measurements were taken after each layer deposition, and the values was converted to the deposited areal mass of MXene and PAA calculated by Sauerbrey equation. The XRD conditions for measurement used Cu K-α radiation with a wavelength of λ = 1.54 Å and photon energy of *E* = 8.04 keV. For microscopy imaging, the Si substrates were scored using a diamond pen then fractured along the score line to prove a clean cross-section and/or to fit on the sample mount for the SEM. Prior to mounting, samples were sputter coated with a thickness of 5 nm to slow film charging due to the electron beam of the SEM. The cross-sectional TEM measurement was performed after peeling a multilayer fragment using the focus ion beam (FIB) milling method with SEM (Tescan FERA-3 model GMH focused ion beam microscope) and then coating platinum on the surface. The small scale *R*_q_ for each LbL multilayer coated on Si wafer was measured by atomic force microscopy (AFM, Bruker Dimension Icon). The gas barrier performances of MXene-PAA LbL composite multilayers were tested by using a Stanford Research Systems residual gas analyzer RGA100 at 10^–6^ Pa. Research grade hydrogen gas was used as inlet gas and the sample multilayers coated onto niobium substrates were heated to 400 °C during the measurements. The electrical conductivity (σ_DC_) was calculated from sheet resistance (*R*_s_) measured by a four-point probe (Lucas Labs S-302-4). The ellipsometric film thicknesses of MXene/PAA LbL multilayers were measured using ellipsometry (Gaertner LSE Stokes ellipsometer) under certain relative humidity (RH) conditions. The environment under certain RH values was controlled in a home-built chamber and was recorded using a humidity sensor (Willhi WH1436H). The error of the humidity sensor was ±1 %. Synchrotron-radiation XPS was performed using surface chemistry experimental station at BL23SU of SPring-8 facility in Japan. The samples for measurement were prepared by coating up to 4-layer pairs onto Si wafer. The source energy was 711.6 eV. The background was subtracted using Shirley type background function and Gaussian–Lorentzian curve function was used to fit all component peaks.

## Supplementary Material















## Notes

Results for MXene and PAA characterization including ζ potential, SEM, FT-IR, XPS, XRD, pH changes, etc; results for MXene/PAA LbL multilayer characterization including film thickness and roughness, UV–vis, QCM, XPS, XRD, morphology analyses, adhesion testing, gas permeance and permeability, synchrotron XPS, electrical conductivity, etc; equations used in this work (PDF)
Movie S1 for tape adhesion testing of MXene/PAA LbL multilayers (MP4)
Movie S2 for tape adhesion testing of MXene/PAA LbL multilayers (MP4)
Movie S3 for tape adhesion testing of MXene/PAA LbL multilayers (MP4)
Movie S4 for tape adhesion testing of MXene/PAA LbL multilayers (MP4)
Movie S5 for tape adhesion testing of MXene/PAA LbL multilayers (MP4)
Movie S6 for tape adhesion testing of MXene/PAA LbL multilayers (MP4)
