# Supplementary material for: Nacre-like MXene/Polyacrylic Acid Layer-by-Layer Multilayers as Hydrogen Gas Barriers
Source: ACS Appl Mater Interfaces. 2025 May 13;17(21):31392–402. doi: 10.1021/acsami.5c03632 (PMC12123567; doi:10.1021/acsami.5c03632)
Supplement: Supplementary file 1 [file am5c03632_si_001.pdf]

Supporting Information

# Nacre-like MXene/polyacrylic acid layer-by-layer multilayers as hydrogen gas barriers

Yang Hyun Auh[1], Natalie N. Neal[2], Kailash Arole[1], Nolan A. Regis[3], Tran Nguyen[1], Shuichi Ogawa[4], Yasutaka Tsuda[5], Akitaka Yoshigoe[5], Miladin Radovic[2], Micah J. Green,[1,2] Hisato Yamaguchi,[*,3] and Jodie L. Lutkenhaus[*,1,2]

[1] Artie McFerrin Department of Chemical Engineering, Texas A&M University, TX 77843, United States of America

[2] Department of Materials Science and Engineering, Texas A&M University, TX 77840, United States of America

[3] Los Alamos National Laboratory, Los Alamos, New Mexico 87545, United States of America,

[4] College of Industrial Technology, Nihon University, 1-2-1 Izumi-cho, Narashino, Chiba 275-8575, Japan,

[5] Materials Sciences Research Center, Japan Atomic Energy Agency, Sayo, Hyogo 679-5148, Japan

Email: **jodie.lutkenhaus@tamu.edu, hyamaguchi@lanl.gov**

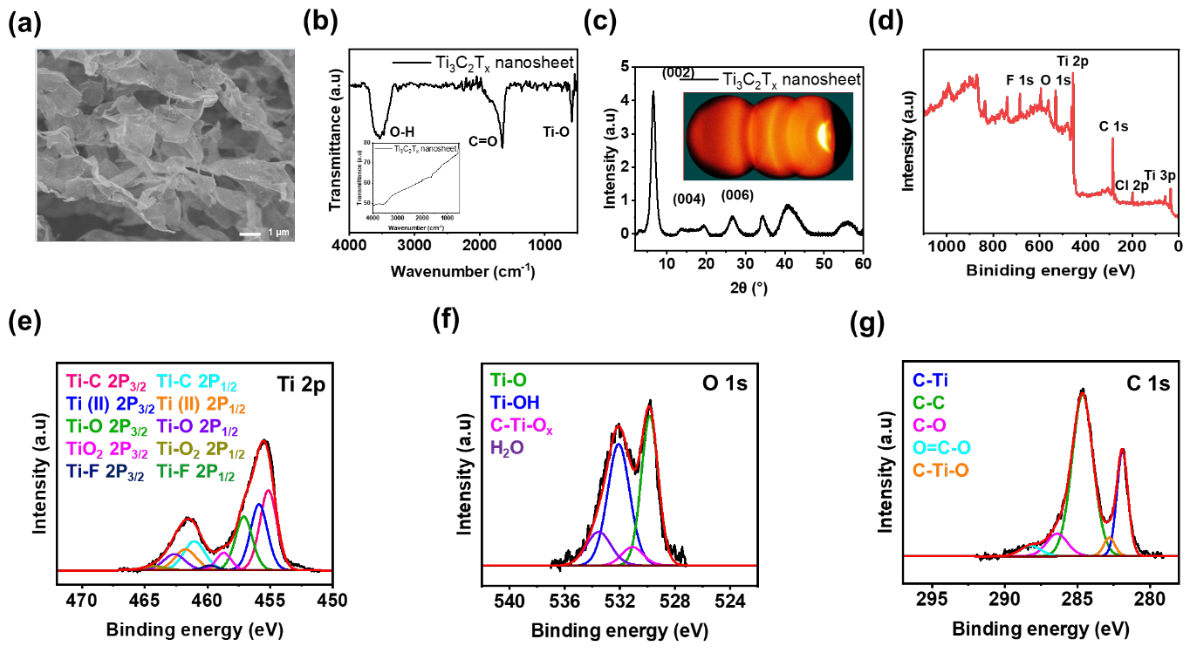


**Figure S1.** (a) SEM images of the exfoliated $Ti_3C_2T_x$ MXene nanosheets. (b) Baseline-subtracted and raw FT-IR spectra of MXene nanosheet film. (c) XRD pattern of MXene nanosheet film and its 2D measurement image. The film was prepared by vacuum-assisted filtration method. (d) Laboratory-based XPS survey scan of MXene nanosheets. The fitted XPS component peaks for (e) Ti 2p, (f) O 1s, and (g) C 1s of MXene nanosheets.

**Table S1.** Laboratory-based XPS analysis results for MXene.

| Element | Element AT (%) | Binding energy (eV) | Component name | Component AT (%) | FWHM |
|---|---|---|---|---|---|
| Ti 2p | 49.8 | 455.2 | Ti-C ($2p_{3/2}$) | 26.1 | 1.6 |
| | | 455.9 | Ti (II) ($2p_{3/2}$) | 21.5 | 1.6 |
| | | 457.1 | Ti-O ($2p_{3/2}$) | 17.5 | 1.6 |
| | | 458.8 | Ti-$O_2$ ($2p_{3/2}$) | 5.0 | 1.4 |
| | | 459.8 | Ti-F ($2p_{3/2}$) | 1.3 | 1.4 |
| | | 461.1 | Ti-C ($2p_{1/2}$) | 11.8 | 2 |
| | | 461.8 | Ti (II) ($2p_{1/2}$) | 8.5 | 2 |
| | | 462.6 | Ti-O ($2p_{1/2}$) | 6.5 | 2 |
| | | 464.2 | Ti-$O_2$ ($2p_{1/2}$) | 1.6 | 2 |
| | | 466.2 | Ti-F ($2p_{1/2}$) | 0.2 | 2 |
| O 1s | 22.3 | 529.8 | Ti-O | 39.3 | 1.5 |
| | | 532.1 | Ti-OH | 41.8 | 1.9 |
| | | 531.1 | C-Ti-$O_x$ | 6.1 | 1.8 |
| | | 533.5 | $H_2O$ | 12.8 | 2.1 |
| C 1s | 27.9 | 281.9 | C-Ti | 21.4 | 0.9 |
| | | 282.5 | C-Ti-O | 3.7 | 0.9 |
| | | 284.6 | C-C | 62.1 | 1.7 |
| | | 286.4 | C-O | 8.7 | 1.7 |
| | | 288.2 | O=C-O | 4.1 | 1.7 |

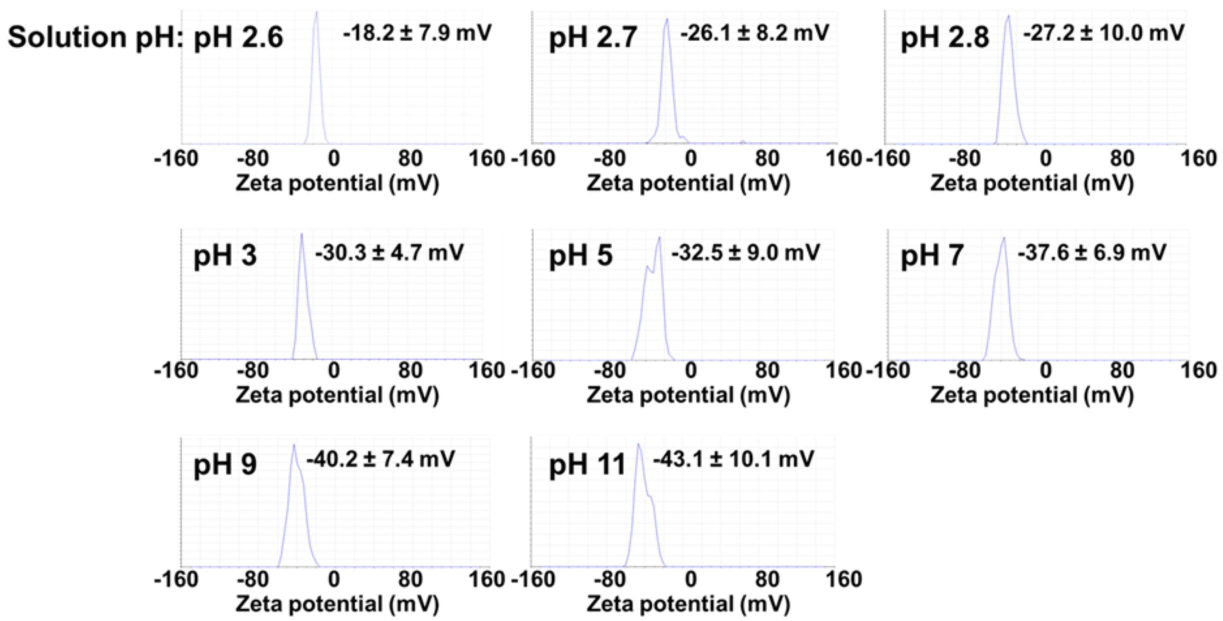


**Figure S2.** Zeta potential of MXene dispersion under different pH conditions.

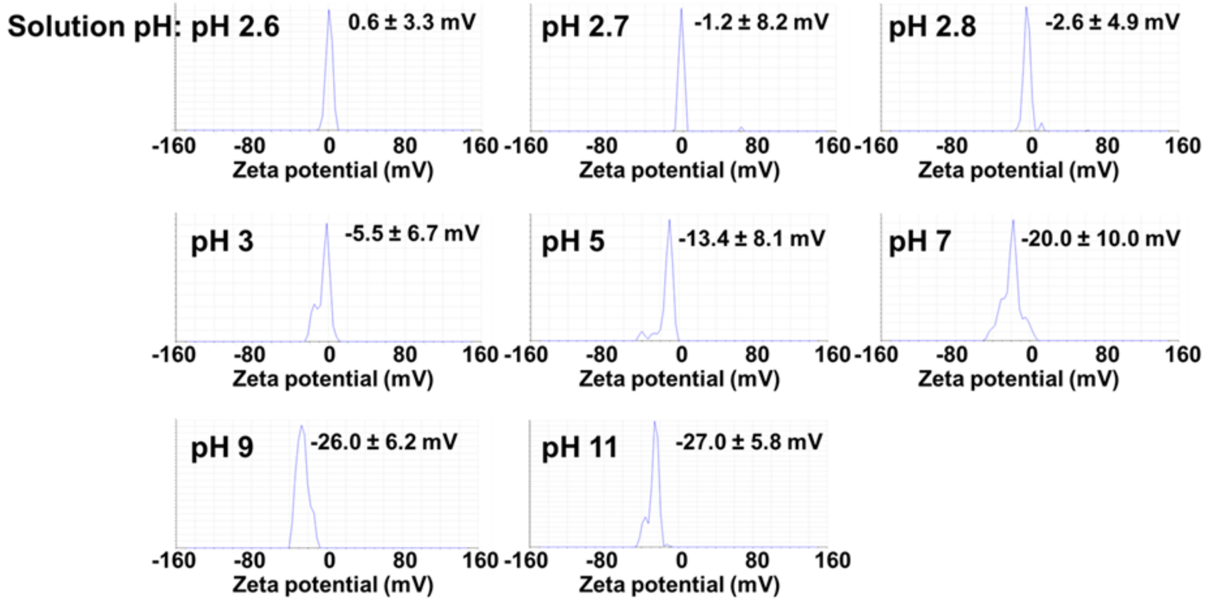


**Figure S3.** Zeta potential of polyacylic acid (PAA) aqueous solutions under different pH conditions.

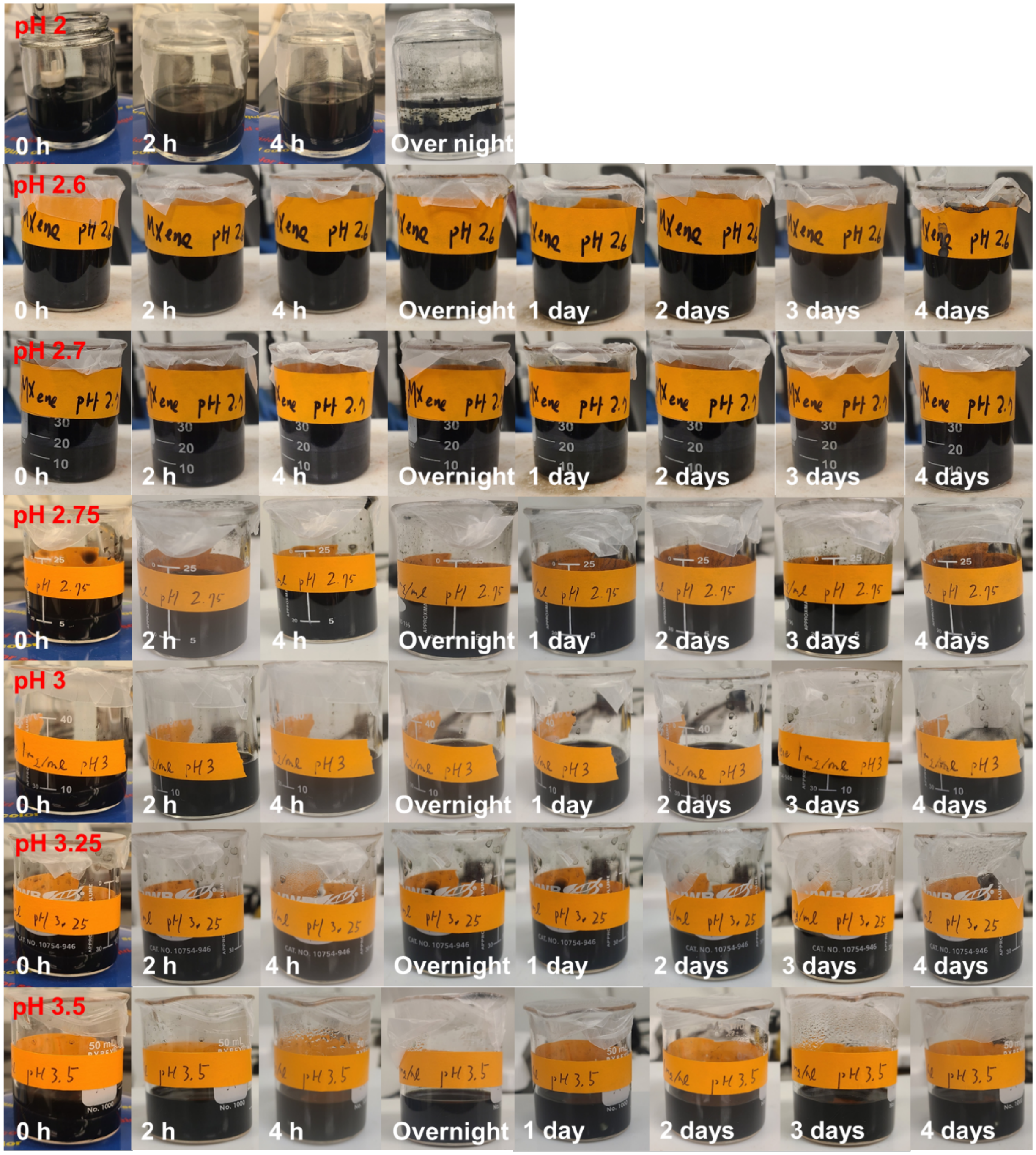


**Figure S4.** Digital images of the changes in each pH-adjusted MXene dispersions over time. The red letters indicate the pH of aqueous solutions.

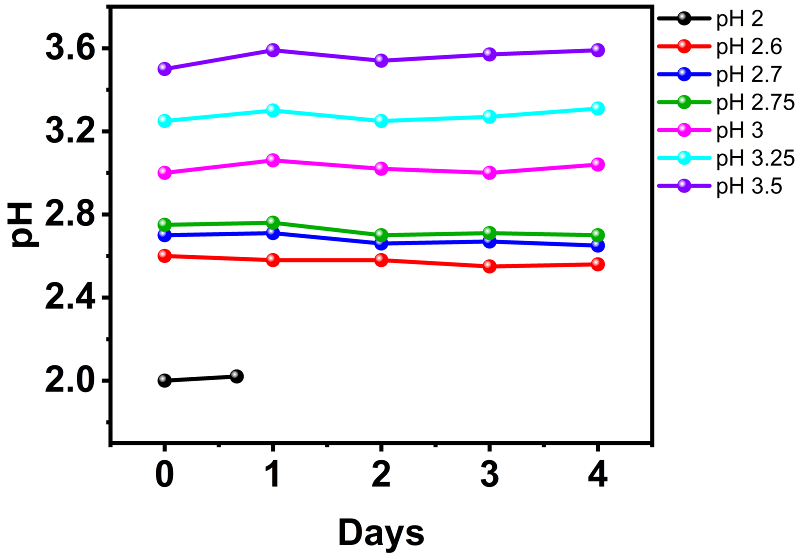


**Figure S5.** Stability of pH-adjusted MXene dispersions.

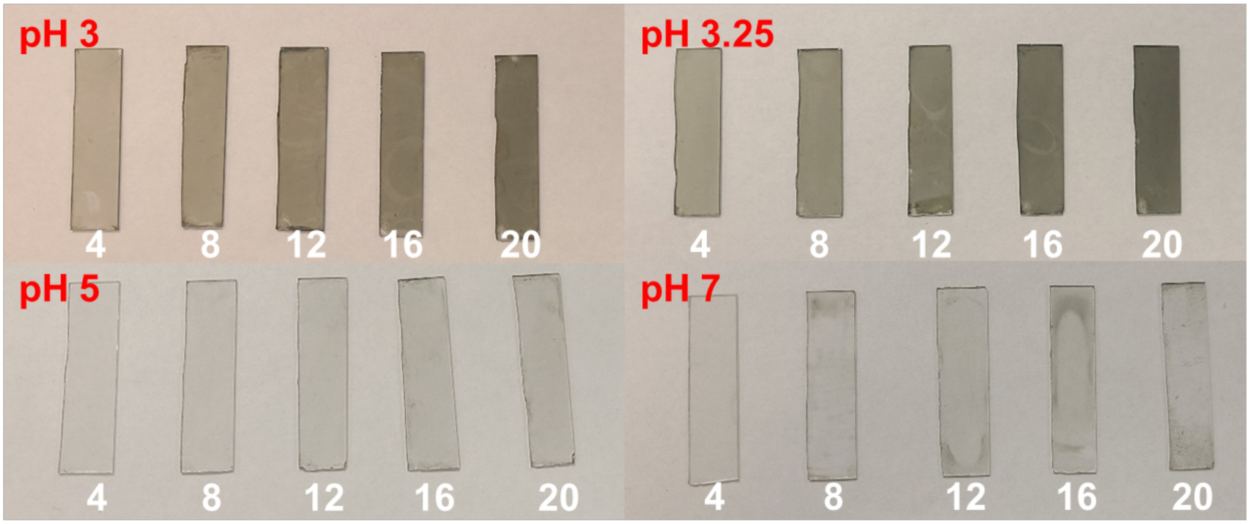


**Figure S6.** Digital images of MXene/PAA LbL multilayers. The red and white letters indicate the film pH and LP number, respectively.

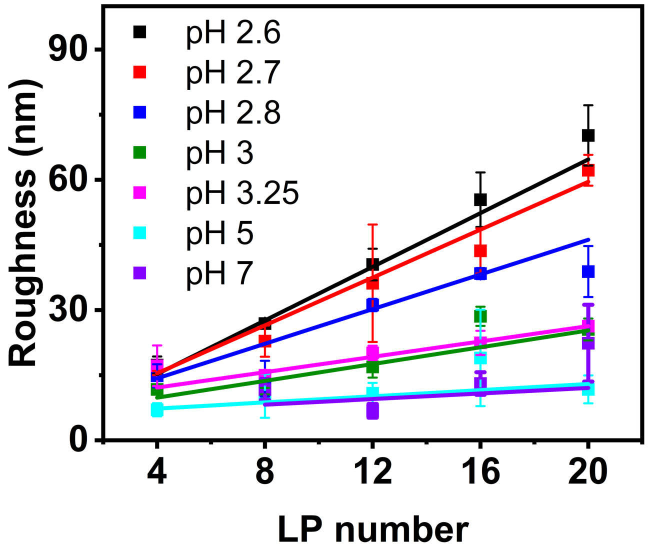


**Figure S7.** Profilometric roughness of pH MXene/PAA LbL multilayers as a function of LP number.

**Table S2.** Rate of profilometric thickness growth of each MXene/PAA LbL multilayer. $\text{Thickness (nm)} = \text{m} \times \text{LP} + \text{n}$

m: Rate of thickness growth

LP: Film layer pair number

n: y-intercept

| **pH** | **m (nm)** | **n (nm)** |
|---|---|---|
| 2.6 | 6.45 | -0.22 |
| 2.7 | 5.03 | 8.27 |
| 2.8 | 3.69 | 11.82 |
| 3 | 2.16 | 12.00 |
| 3.25 | 1.86 | 12.56 |
| 5 | 0.54 | 8.91 |
| 7 | 0.48 | 9.18 |

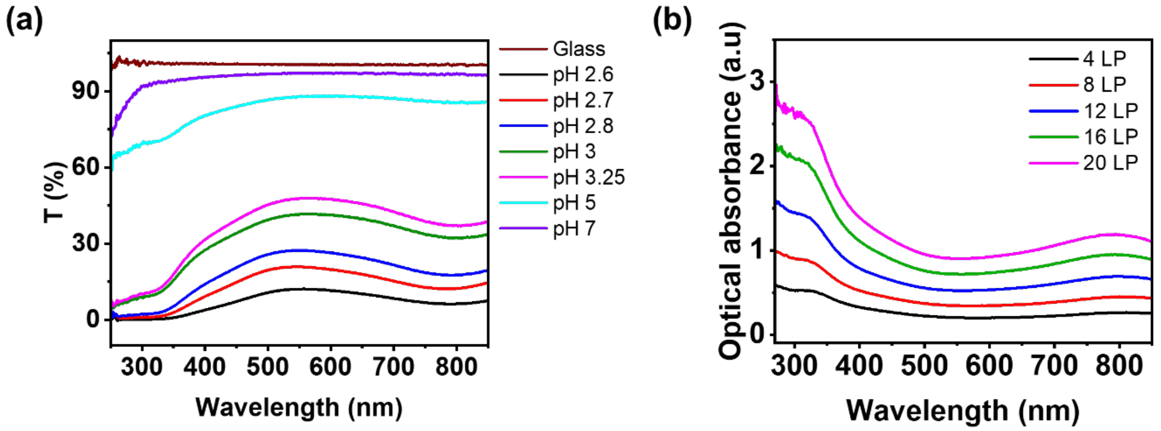


**Figure S8.** (a) Optical transmittance of different pH MXene/PAA films. (b) Optical absorbance spectra of pH 2.6 LbL multilayers with different LP number.

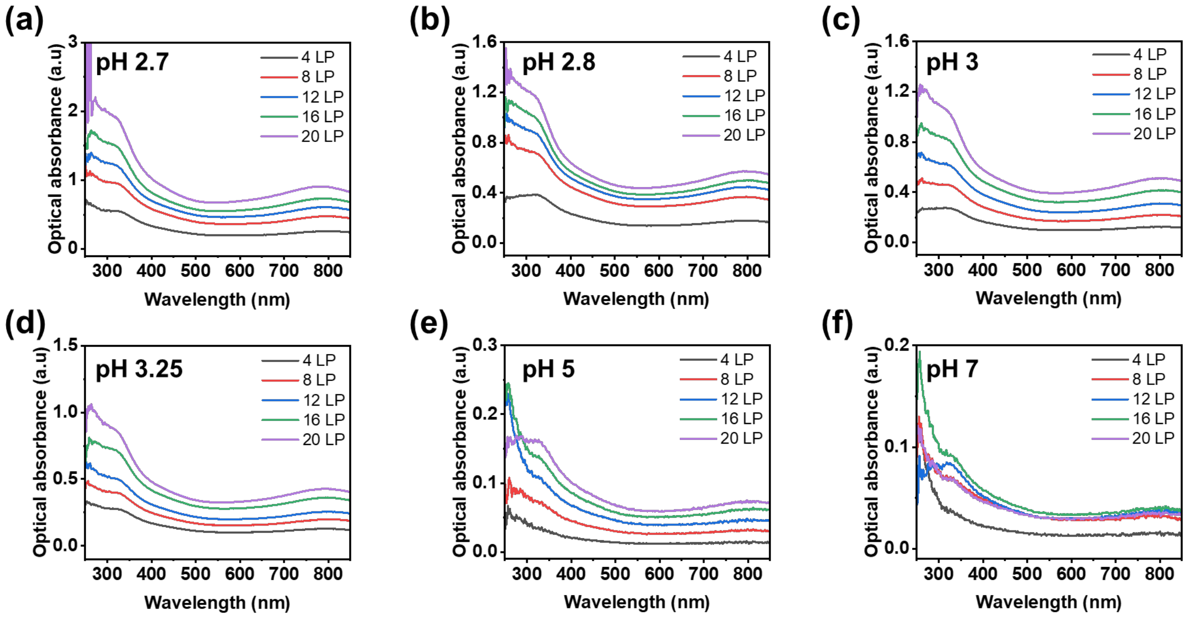


**Figure S9.** Optical absorbance spectra of (a) pH2.7, (b) pH 2.8, (c) pH 3, (d) pH 3.25, (e) pH 5, and (f) pH 7 LbL multilayer with different LP number.

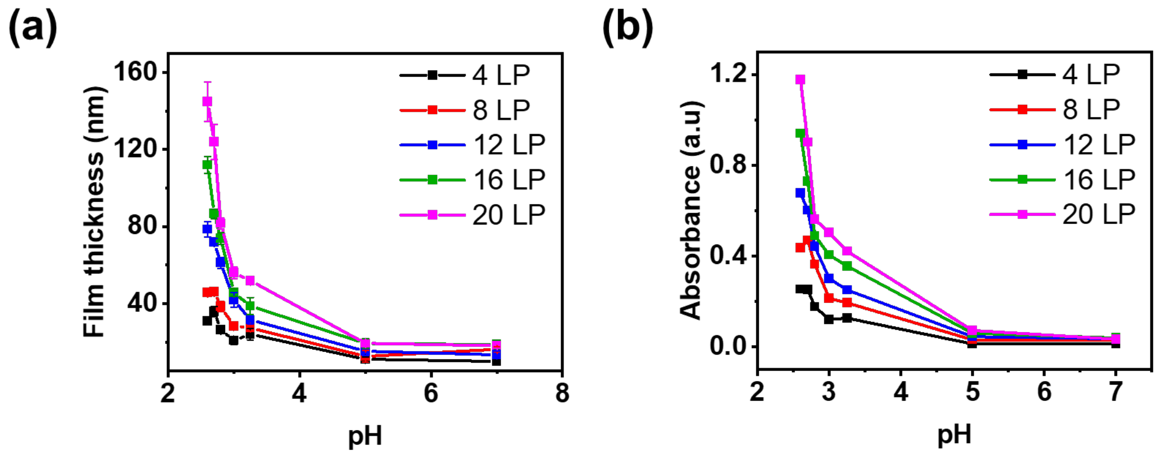


**Figure S10.** (a) Profilometric film thickness, and (b) optical absorbance at 770 nm of MXene/PAA LbL multilayers as a function of film pH with different LP numbers.

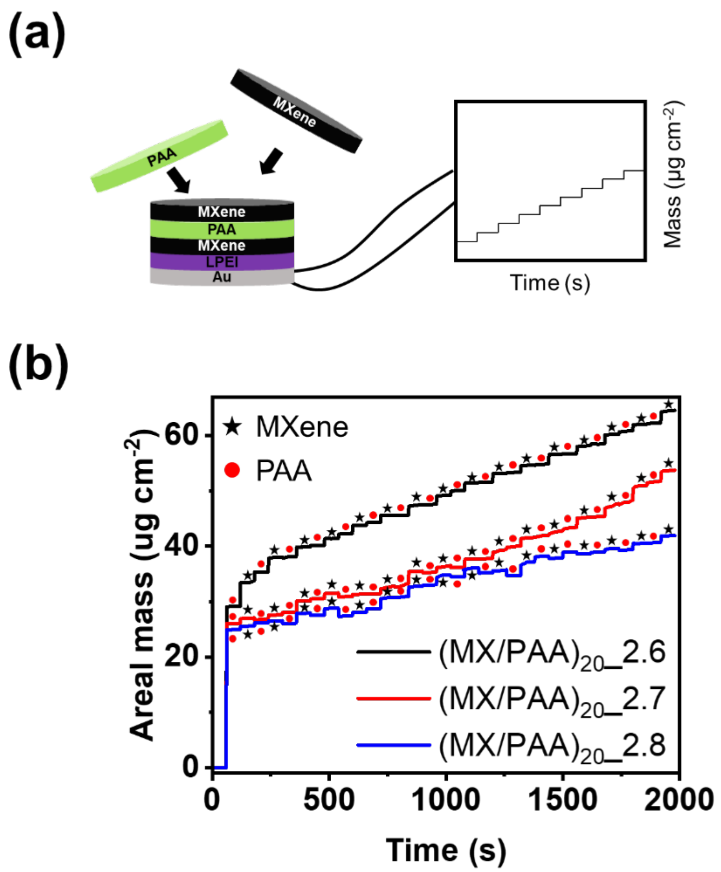


**Figure S11.** (a) Illustration for the QCM measurement. (b) Areal mass growth of the MXene/PAA LbL multilayers as a function of time. The black star and red circle shaped markers indicate the MXene and PAA layers, respectively.

The rate of areal mass growth of MXene/PAA LbL multilayers under different pH is shown below.

$m_{2.6}$ ($\mu g\ cm^{-2}$) = 2.0 × $\mu g\ cm^{-2}\ LP^{-1}$ + 24.9 ($R^2$: 0.99)

$m_{2.7}$ ($\mu g\ cm^{-2}$) = 1.7 × $\mu g\ cm^{-2}\ LP^{-1}$ + 17.1 ($R^2$: 0.97)

$m_{2.8}$ ($\mu g\ cm^{-2}$) = 1.1 × $\mu g\ cm^{-2}\ LP^{-1}$ + 19.7 ($R^2$: 0.98)

where $m_{2.6,\ 2.7,\ or\ 2.8}$ indicates the areal mass of MXene/PAA LbL multilayers deposited under each pH.

**Table S3.** Laboratory-based XPS analysis results for pH 2.6 LbL multilayer.

| Element | Element AT (%) | Binding energy (eV) | Component name | Component AT (%) | FWHM |
|---|---|---|---|---|---|
| Ti 2p | 31.9 | 455.2 | Ti-C ($2p_{3/2}$) | 23.8 | 1.6 |
| | | 455.9 | Ti (II) ($2p_{3/2}$) | 18.9 | 1.6 |
| | | 457.1 | Ti-O ($2p_{3/2}$) | 16.1 | 1.6 |
| | | 458.7 | $TiO_2$ ($2p_{3/2}$) | 9.6 | 1.4 |
| | | 459.5 | Ti-F ($2p_{3/2}$) | 1.7 | 1.4 |
| | | 461 | Ti-C ($2p_{1/2}$) | 12.7 | 2 |
| | | 461.9 | Ti (II) ($2p_{1/2}$) | 8.1 | 2 |
| | | 462.8 | Ti-O ($2p_{1/2}$) | 5.0 | 2 |
| | | 464.4 | $TiO_2$ ($2p_{1/2}$) | 3.5 | 2 |
| | | 466.1 | Ti-F ($2p_{1/2}$) | 0.6 | 2 |
| O 1s | 42.5 | 529.8 | Ti-O | 24.3 | 1.5 |
| | | 531.9 | Ti-OH | 26.4 | 1.9 |
| | | 531 | C-Ti-$O_x$ | 4.6 | 1.8 |
| | | 531.7 | O=C-O | 20.6 | 2.6 |
| | | 533.2 | C-O | 14.6 | 2.6 |
| | | 533.2 | $H_2O$ | 9.5 | 2.1 |
| C 1s | 25.6 | 281.5 | C-Ti | 10.2 | 0.9 |
| | | 282.5 | C-Ti-O | 2.4 | 0.9 |
| | | 284.7 | C-C | 49.8 | 1.7 |
| | | 286.4 | C-O | 16.8 | 1.7 |
| | | 288.5 | O=C-O | 20.8 | 1.7 |

**Table S4.** Laboratory-based XPS analysis results for pH 2.7 LbL multilayer.

| Element | Element AT (%) | Binding energy (eV) | Component name | Component AT (%) | FWHM |
|---|---|---|---|---|---|
| Ti 2p | 41.7 | 455.1 | Ti-C ($2p_{3/2}$) | 26.3 | 1.6 |
| | | 456 | Ti (II) ($2p_{3/2}$) | 19.6 | 1.6 |
| | | 457.2 | Ti-O ($2p_{3/2}$) | 12.5 | 1.6 |
| | | 458.7 | $TiO_2$ ($2p_{3/2}$) | 10.8 | 1.4 |
| | | 459.5 | Ti-F ($2p_{3/2}$) | 1.8 | 1.4 |
| | | 460.9 | Ti-C ($2p_{1/2}$) | 13.7 | 2 |
| | | 461.9 | Ti (II) ($2p_{1/2}$) | 7.1 | 2 |
| | | 462.8 | Ti-O ($2p_{1/2}$) | 4.4 | 2 |
| | | 464.3 | $TiO_2$ ($2p_{1/2}$) | 3.6 | 2 |
| | | 466.1 | Ti-F ($2p_{1/2}$) | 0.2 | 2 |
| O 1s | 34.5 | 529.7 | Ti-O | 22.0 | 1.5 |
| | | 531.8 | Ti-OH | 30.8 | 1.9 |
| | | 531 | C-Ti-$O_x$ | 6.3 | 1.8 |
| | | 531.7 | O=C-O | 19.0 | 2.6 |
| | | 533.2 | C-O | 12.8 | 2.6 |
| | | 533.2 | $H_2O$ | 9.1 | 2.1 |
| C 1s | 23.8 | 281.6 | C-Ti | 14.0 | 0.9 |
| | | 282.5 | C-Ti-O | 2.6 | 0.9 |
| | | 284.7 | C-C | 52.2 | 1.7 |
| | | 286.4 | C-O | 14.9 | 1.7 |
| | | 288.5 | O=C-O | 16.3 | 1.7 |

**Table S5.** Laboratory-based XPS analysis results for pH 2.8 LbL multilayer.

| Element | Element AT (%) | Binding energy (eV) | Component name | Component AT (%) | FWHM |
|---|---|---|---|---|---|
| Ti 2p | 47.0 | 455.1 | Ti-C ($2p_{3/2}$) | 25.6 | 1.6 |
| | | 455.9 | Ti (II) ($2p_{3/2}$) | 19.7 | 1.6 |
| | | 457.1 | Ti-O ($2p_{3/2}$) | 13.9 | 1.6 |
| | | 458.7 | $TiO_2$ ($2p_{3/2}$) | 9.6 | 1.4 |
| | | 459.5 | Ti-F ($2p_{3/2}$) | 2.1 | 1.4 |
| | | 461 | Ti-C ($2p_{1/2}$) | 13.3 | 2 |
| | | 461.9 | Ti (II) ($2p_{1/2}$) | 7.5 | 2 |
| | | 462.8 | Ti-O ($2p_{1/2}$) | 5.1 | 2 |
| | | 464.3 | $TiO_2$ ($2p_{1/2}$) | 3.0 | 2 |
| | | 466.1 | Ti-F ($2p_{1/2}$) | 0.2 | 2 |
| O 1s | 30.6 | 529.7 | Ti-O | 25.5 | 1.5 |
| | | 531.8 | Ti-OH | 35.0 | 1.9 |
| | | 531.1 | C-Ti-$O_x$ | 6.1 | 1.8 |
| | | 531.7 | O=C-O | 15.2 | 2.6 |
| | | 533.2 | C-O | 9.0 | 2.6 |
| | | 533.2 | $H_2O$ | 9.3 | 2.1 |
| C 1s | 22.4 | 281.6 | C-Ti | 16.6 | 0.9 |
| | | 282.6 | C-Ti-O | 3.0 | 0.9 |
| | | 284.6 | C-C | 52.6 | 1.7 |
| | | 286.3 | C-O | 15.4 | 1.7 |
| | | 288.5 | O=C-O | 12.4 | 1.7 |

In O 1s and C 1s (**Figure 3b-d** and **Table S3** — **S5**), LbL multilayers showed a clear increase of carboxyl groups compared to bare MXene (**Figure S1f,g**). In O 1s (**Figure 3b-d** and **Table S3** — **S5**), pH 2.6 LbL multilayer exhibited the highest ratio of carboxyl groups originating from PAA (O-C=O: 20.6 %, and C-O: 12.2 %). Compared to pH 2.7 (C-O: 14.9 %, and O-C=O: 16.3 %) and 2.8 (C-O: 15.4 %, and O-C=O: 12.4 %) LbL multilayers, the pH 2.6 LbL multilayer exhibited the highest peak ratio for the C-O and O-C=O chemical bonds (16.8 %, and 20.8 %, respectively) in C 1s spectrum (**Figure 3b-d)**, consistent with the trend observed in O 1s results. The elevated carboxyl group content at higher film pH is likely due to a larger amount of PAA interacting with MXenes by hydrogen bonding within the multilayer.

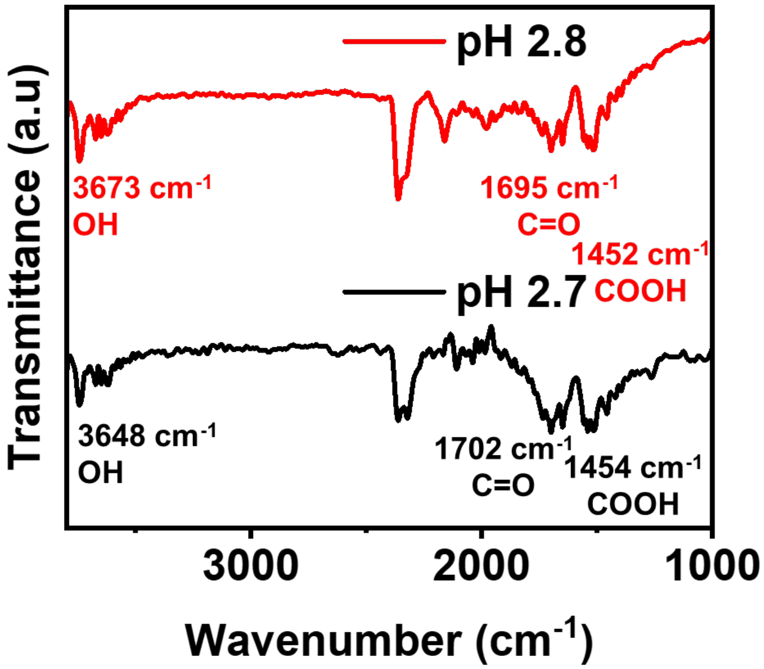


**Figure S12.** FT-IR spectra of pH 2.7 and pH 2.8 MXene/PAA LbL multilayers.

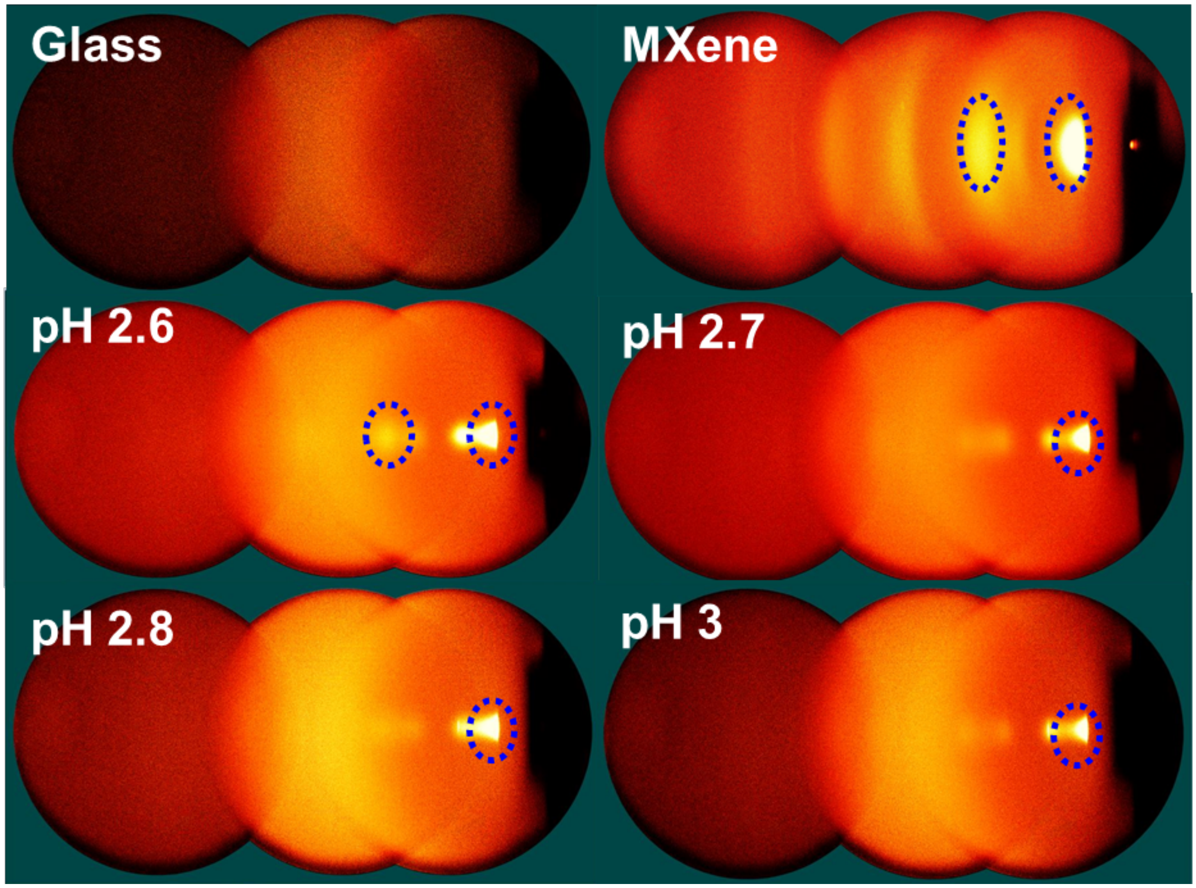


**Figure S13.** XRD 2D images of the glass substrate, MXene, and LbL multilayers.

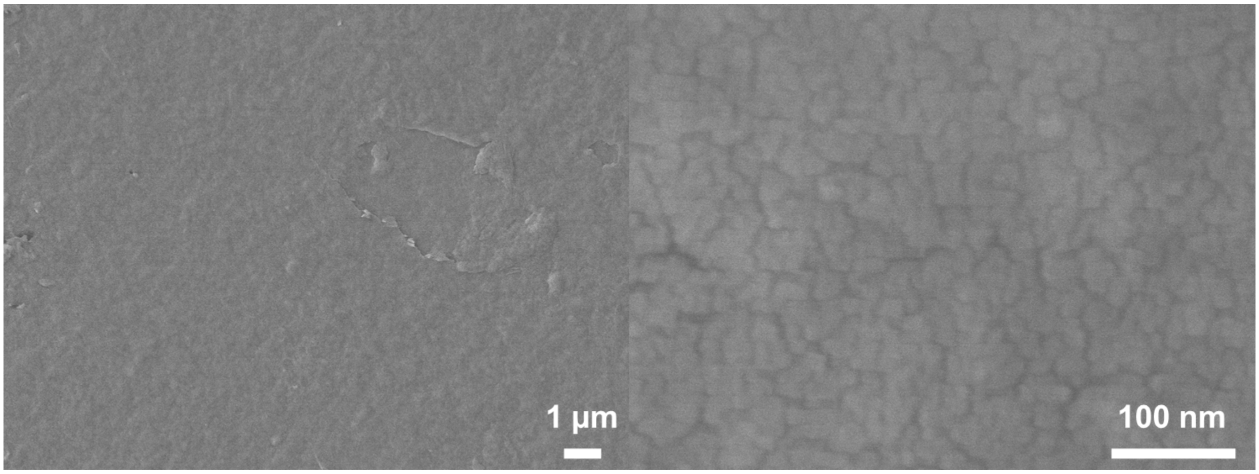


**Figure S14.** Top-view images of pH 2.6 LbL multilayer with different scales and regions.

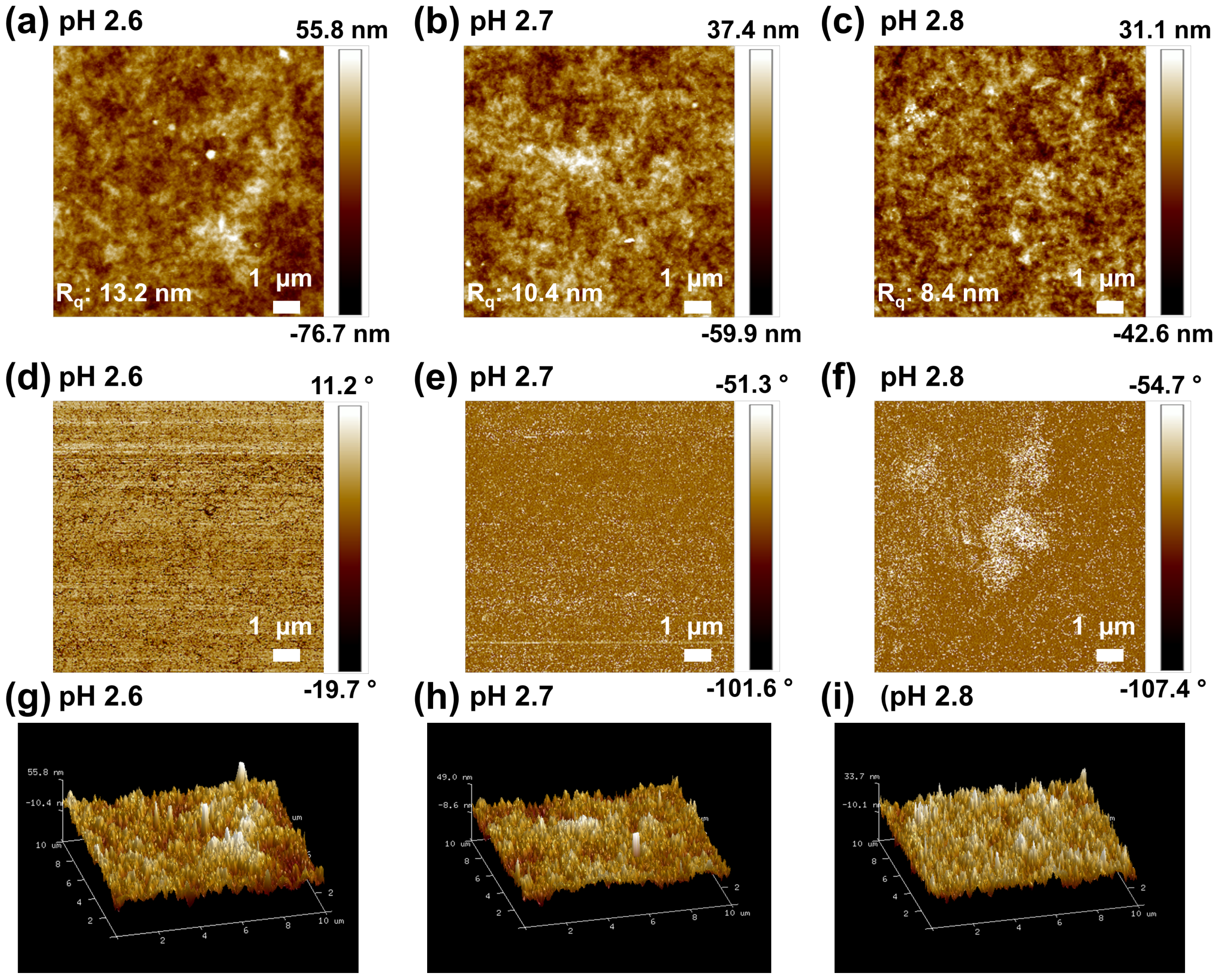


**Figure S15.** (a-c) Surface morphology, (d-f) phase images, and (g-i) 3D surface morphology of pH 2.6, 2.7, and 2.8 LbL multilayers.

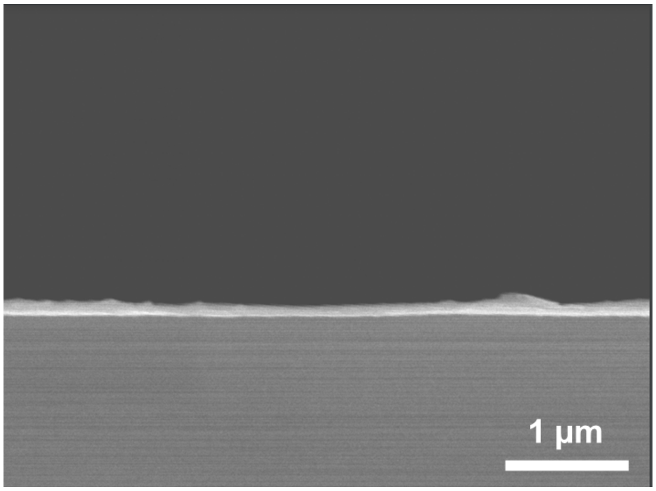


**Figure S16.** Cross-sectional SEM image of pH 2.6 LbL multilayer in 1 μm scale.

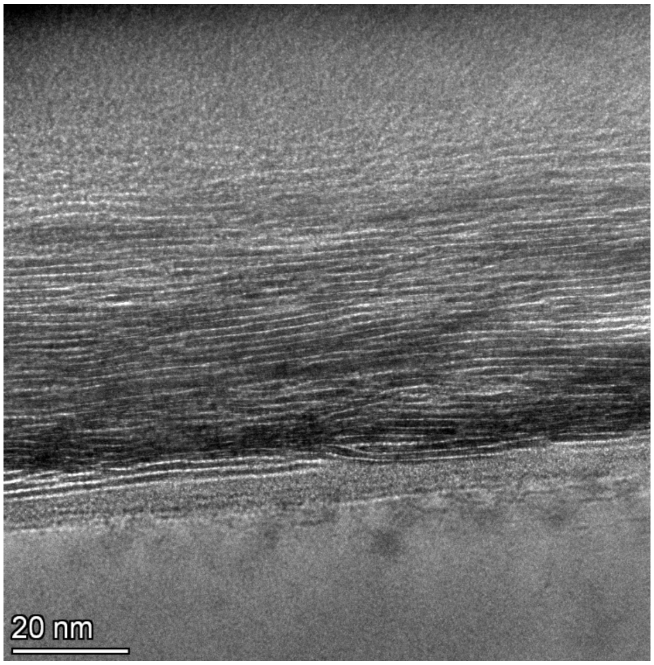


**Figure S17.** Cross-sectional TEM images of pH 2.6 LbL multilayer in 20 nm scale.

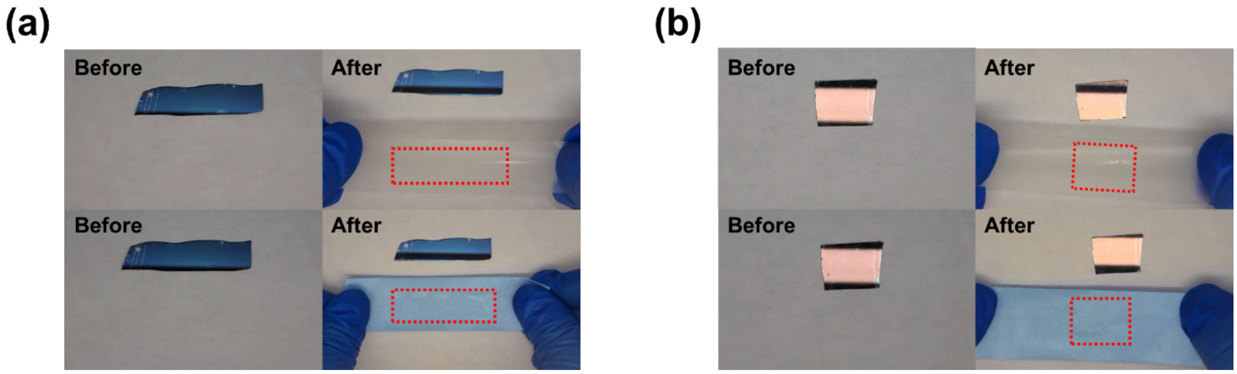


**Figure S18.** Digital images of adhesion test for (a) pH 2.7 and (b) 2.8 LbL multilayers.

**Table S6.** Gas barrier properties of MXene/PAA LbL multilayers and bare niobium substrate.

| | pH 2.6 | pH 2.7 | pH 2.8 | Nb |
|---|---|---|---|---|
| [a]$P_{saturation}$ (Pa) | $3.68\times10^{-7}$ ± $5.11\times10^{-6}$ | $2.05\times10^{-5}$ ± $1.36\times10^{-6}$ | $1.19\times10^{-5}$ ± $1.09\times10^{-6}$ | 3.50×10-5 ± $1.49\times10^{-6}$ |
| [b]Leakage rate ($m^3\cdot atm\cdot s^{-1}$) | $1.03\times10^{-13}$ ± $2.32\times10^{-12}$ | $5.98\times10^{-12}$ ± $1.38\times10^{-12}$ | $3.48\times10^{-12}$ ± $2.40\times10^{-12}$ | $1.02\times10^{-11}$ ± $1.77\times10^{-13}$ |
| [c]$Permeance_{film+substrate}$ ($m^3\cdot m^{-2}\cdot atm^{-1}\cdot day^{-1}$) | $4.04\times10^{-4}$ ± $1.26\times10^{-3}$ | $2.33\times10^{-2}$ ± $1.55\times10^{-3}$ | 1.36×10-2 ± $1.24\times10^{-3}$ | $3.98\times10^{-2}$ ± $1.70\times10^{-3}$ |
| [d]$Permeance_{film}$ ($m^3\cdot m^{-2}\cdot atm^{-1}\cdot day^{-1}$) | $4.08\times10^{-4}$ ± $1.40\times10^{-3}$ | $5.63\times10^{-2}$ ± $7.93\times10^{-3}$ | $2.06\times10^{-2}$ ± $2.69\times10^{-3}$ | - |
| [e]$Permeability_{film+substrate}$ ($m^3\cdot m\cdot m^{-2}\cdot atm^{-1}\cdot day^{-1}$) | $5.13\times10^{-8}$ ± $1.61\times10^{-7}$ | $2.96\times10^{-6}$ ± $1.97\times10^{-7}$ | $1.73\times10^{-6}$ ± $1.58\times10^{-7}$ | $5.06\times10^{-6}$ ± $2.16\times10^{-7}$ |
| [d]$Permeability_{film}$ ($m^3\cdot m\cdot m^{-2}\cdot atm^{-1}\cdot day^{-1}$) | $1.32\times10^{-11}$ ± $4.52\times10^{-11}$ | $1.57\times10^{-9}$ ± $2.21\times10^{-10}$ | $4.91\times10^{-10}$ ± $6.41\times10^{-11}$ | - |

[a]Saturated pressure of each LbL multilayer. [b]Calibrated leakage rate of each LbL multilayer. [c]Permeance of film-coated substrate calculated using leakage rate and saturated pressure of each LbL multilayer. [d]Permeance of coated film calculated using leakage rate and saturated pressure of each LbL multilayer. [e]Permeability of film-coated substrate of each LbL multilayer. [f]Permeability of each coated LbL multilayer.

**Table S7.** Comparison of film thickness and permeability for MXene/PAA LbL multilayers and other conventional composite films. The Nb substrate and MXene LbL multilayers used in this work are highlighted in bold.

| Sample | Sample name | [a]l (nm) | [b]Permeability ($cc \cdot mm \cdot day^{-1} \cdot m^{-2} \cdot MPa^{-1}$) | Reference No. | Coating method |
|---|---|---|---|---|---|
| A | MWCNT/PMMA | $3.00\times10^{4}$ | $1.94\times10^{5}$ | 1 | Solution casting |
| **Nb** | **Nb** | **1.27**$\times10^{5}$ | **$4.99\times10^{4}$ ± $2.13\times10^{3}$** | - | - |
| B | Nyon 6 | $3.84\times10^{6}$ | $6.98\times10^{2}$ | 2 | - |
| C | FAS-CeO2@GFs/EP 40 wt% | $6.17\times10^{4}$ | $6.47\times10^{2}$ | 3 | Doctor blading |
| **pH 2.7** | **pH 2.7** | **31.0** | **17.2 ± 1.4** | **This work** | **LbL** |
| **pH 2.8** | **pH 2.8** | **26.5** | **5.39 ± 0.44** | **Thir work** | **LbL** |
| D | 60% mGO-ODA/MAPP | $2.44\times10^{4}$ | 2.56 | 4 | Spray coating |
| E | 1 f-GO-BN/S-IPN | $1.19\times10^{4}$ | 0.69 | 2 | Solvent casting |
| F | 10 f-MXene-GO/EAA | $1.30\times10^{4}$ | 0.29 | 5 | Spray coating |
| G | 2 f-GO-BN/S-IPN | $1.21\times10^{4}$ | 0.20 | 2 | Solvent casting |
| H | (PEI/GO0.05)20 | 98.0 | 0.15 | 6 | LbL |
| **pH 2.6** | **pH 2.6** | **35.8** | **0.14 ± 0.01** | **This work** | **LbL** |
| I | 5 f-GO-BN/S-IPN | $1.17\times10^{4}$ | 0.11 | 2 | Solvent casting |
| J | 10 f-GO-BN/S-IPN | $1.25\times10^{4}$ | 0.06 | 2 | Solvent casting |
| K | PSPG2.0 | $2.60\times10^{2}$ | 0.03 | 7 | LbL |
| L | PSS-RGO/PEI-RGO | $2.70\times10^{2}$ | 0.02 | 8 | LbL |

[a]Film thickness. [b]Film permeability of Nb substrate, MXene/PAA LbL multilayers and conventional reported membranes.

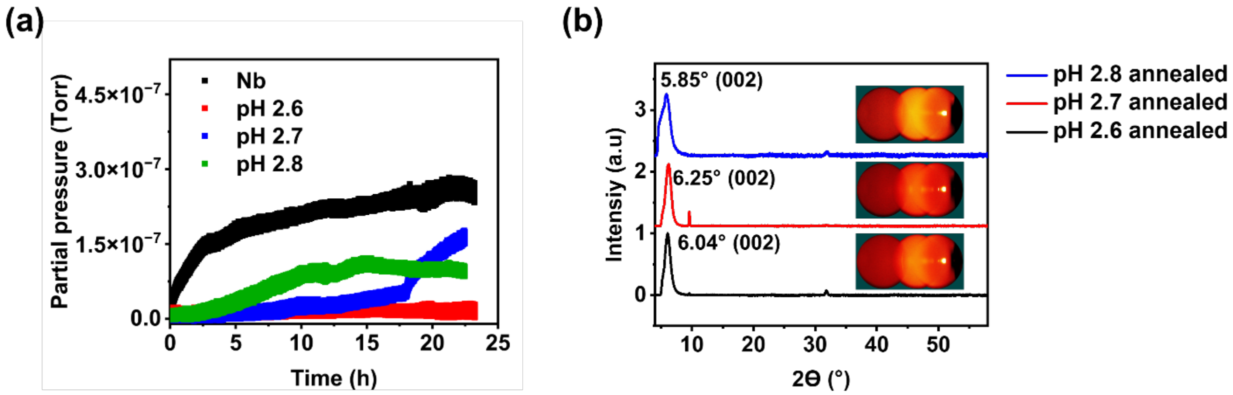


**Figure S19.** (a) Time evolution of hydrogen partial pressure through bare niobium substrate and those coated with MXene/PAA LbL multilayers (b) XRD patterns and 2D images of pH 2.6, 2.7, and 2.8 LbL multilayers after heating at 400 °C under Ar atmosphere. Ar gas was used to prevent MXene oxidation.

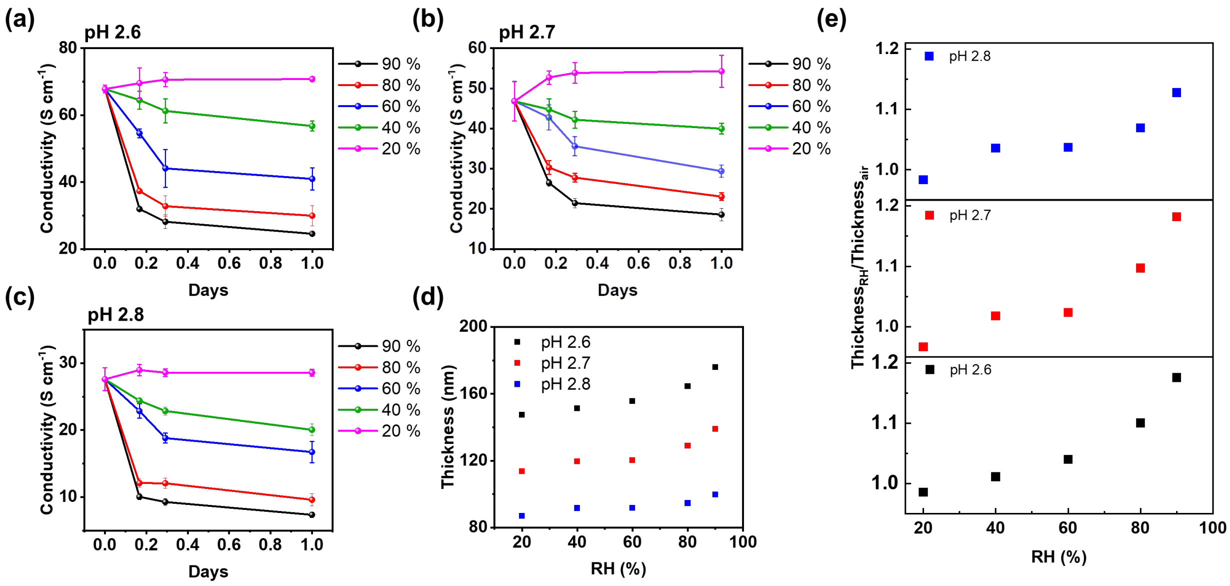


**Figure S20.** Electrical conductivity ($\sigma_{DC}$) changes in (a) pH 2.6, (b) pH 2.7, and (c) pH 2.8 MXene/PAA LbL multilayers over time under different relative humidity (RH) conditions. (d) Ellipsometric thickness changes in pH 2.6, pH 2.7, and pH 2.8 MXene/PAA LbL multilayers as a function of RH. (e) Normalized ellipsometric film thickness ($Thickness_{RH}/Thickness_{air}$) as a function of RH. The normalized thickness changes of the multilayer coated on the Si substrate under specific RH conditions.

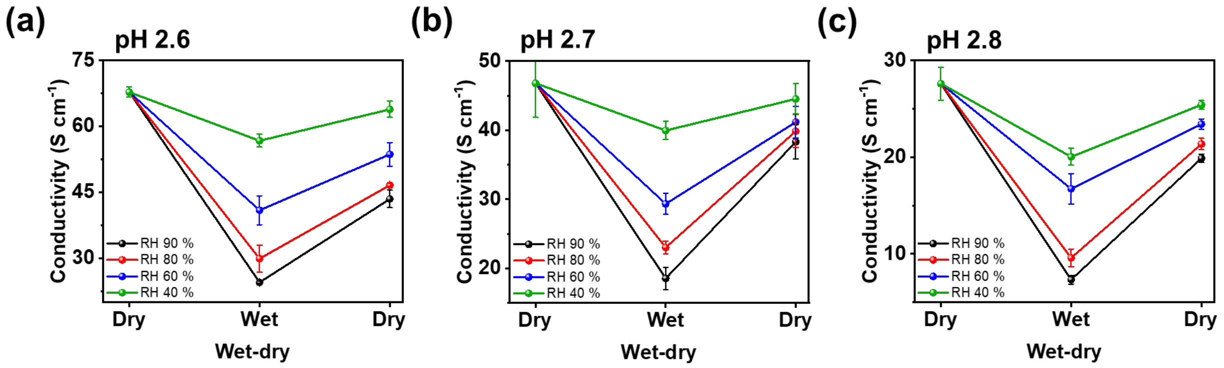


**Figure S21.** Electrical changes of (a) pH 2.6, (b) pH 2.7, and (c) pH 2.8 MXene/PAA LbL multilayers after exposure to the different RH environments for 1 day and subsequent drying under RH conditions of air for 1 day.

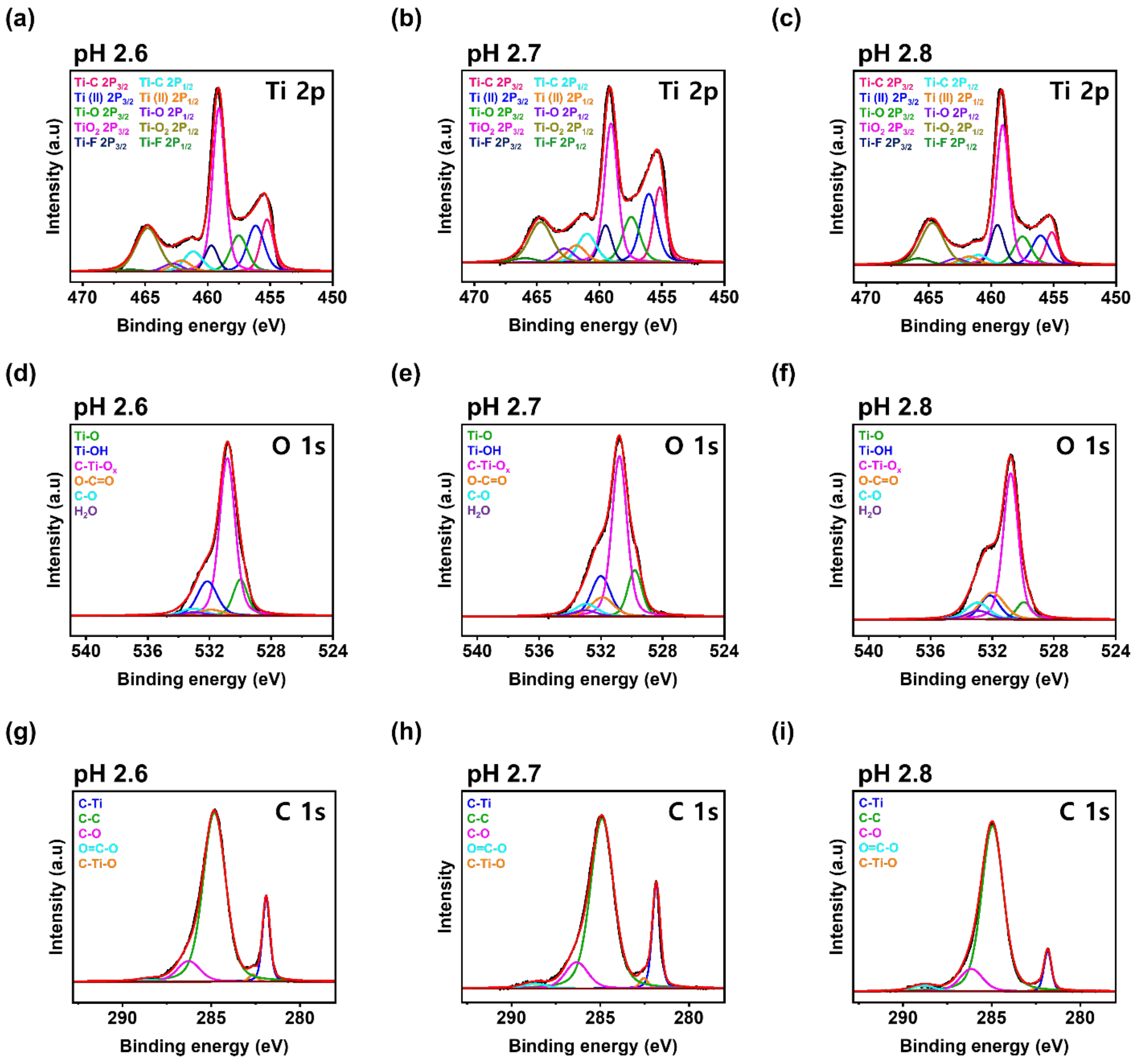


**Figure S22.** XPS component peak fittings for (a-c) Ti 2p, (d-f) O 1s, and (g-i) C 1s of each LbL multilayer measured using SR-XPS.

**Table S8.** SR-XPS analysis results for pH 2.6 LbL multilayer.

| Element | Element AT (%) | Binding energy (eV) | Component name | Component AT (%) | FWHM |
|---|---|---|---|---|---|
| Ti 2p | 54.4 | 455.3 | Ti-C ($2P_{3/2}$) | 10.4 | 1.2 |
| | | 456.1 | Ti (II) ($2P_{3/2}$) | 12.4 | 1.6 |
| | | 457.4 | Ti-O ($2P_{3/2}$) | 9.8 | 1.6 |
| | | 459 | $TiO_2$ | 33.0 | 1.2 |
| | | 459.7 | Ti-F ($2P_{3/2}$) | 5.3 | 1.3 |
| | | 461.1 | Ti-C ($2P_{1/2}$) | 6.0 | 1.8 |
| | | 462.1 | Ti (II) ($2P_{1/2}$) | 3.4 | 1.8 |
| | | 462.8 | Ti-O ($2P_{1/2}$) | 2.8 | 1.8 |
| | | 464.7 | $TiO_2$ ($2P_{1/2}$) | 16.1 | 2.2 |
| | | 466.1 | Ti-F ($2P_{1/2}$) | 0.8 | 2.2 |
| O 1s | 24.7 | 530 | Ti-O | 12.9 | 1.1 |
| | | 532.1 | Ti-OH | 17.0 | 1.5 |
| | | 530.8 | C-Ti-$O_x$ | 62.0 | 1.2 |
| | | 531.9 | O=C-O | 3.6 | 1.8 |
| | | 533 | C-O | 2.0 | 1.8 |
| | | 533.1 | $H_2O$ | 2.5 | 1.8 |
| C 1s | 20.9 | 281.8 | C-Ti | 12.2 | 0.5 |
| | | 282.7 | C-Ti-O | 1.1 | 0.5 |
| | | 284.8 | C-C | 76.5 | 1.5 |
| | | 286.3 | C-O | 9.4 | 1.5 |
| | | 288.5 | O=C-O | 0.8 | 1.5 |

**Table S9.** SR-XPS analysis results for pH 2.7 LbL multilayer.

| Element | Element AT (%) | Binding energy (eV) | Component name | Component AT (%) | FWHM |
|---|---|---|---|---|---|
| Ti 2p | 50.9 | 455.2 | Ti-C ($2P_{3/2}$) | 12.6 | 1.2 |
| | | 456 | Ti (II) ($2P_{3/2}$) | 15.5 | 1.6 |
| | | 457.4 | Ti-O ($2P_{3/2}$) | 10.9 | 1.6 |
| | | 459 | $TiO_2$ | 25.2 | 1.2 |
| | | 459.5 | Ti-F ($2P_{3/2}$) | 6.2 | 1.3 |
| | | 461 | Ti-C ($2P_{1/2}$) | 7.3 | 1.8 |
| | | 461.8 | Ti (II) ($2P_{1/2}$) | 4.5 | 1.8 |
| | | 462.8 | Ti-O ($2P_{1/2}$) | 3.6 | 1.8 |
| | | 464.6 | $TiO_2$ ($2P_{1/2}$) | 12.8 | 2.2 |
| | | 466 | Ti-F ($2P_{1/2}$) | 1.4 | 2.2 |
| O 1s | 21.8 | 529.8 | Ti-O | 13.9 | 1.1 |
| | | 532 | Ti-OH | 16.3 | 1.5 |
| | | 530.8 | C-Ti-$O_x$ | 51.3 | 1.2 |
| | | 531.9 | O=C-O | 9.3 | 1.8 |
| | | 533 | C-O | 6.1 | 1.8 |
| | | 533 | $H_2O$ | 3.1 | 1.8 |
| C 1s | 27.3 | 281.8 | C-Ti | 15.1 | 0.5 |
| | | 282.5 | C-Ti-O | 1.5 | 0.5 |
| | | 284.9 | C-C | 70.7 | 1.5 |
| | | 286.3 | C-O | 10.7 | 1.5 |
| | | 288.5 | O=C-O | 2.0 | 1.5 |

**Table S10.** SR-XPS analysis results for pH 2.8 LbL multilayer.

| Element | Element AT (%) | Binding energy (eV) | Component name | Component AT (%) | FWHM |
|---|---|---|---|---|---|
| Ti 2p | 43.0 | 455.2 | Ti-C ($2P_{3/2}$) | 7.5 | 1.2 |
| | | 456 | Ti (II) ($2P_{3/2}$) | 9.4 | 1.6 |
| | | 457.4 | Ti-O ($2P_{3/2}$) | 9.0 | 1.6 |
| | | 459 | $TiO_2$ | 34.6 | 1.2 |
| | | 459.5 | Ti-F ($2P_{3/2}$) | 9.8 | 1.3 |
| | | 461 | Ti-C ($2P_{1/2}$) | 3.5 | 1.8 |
| | | 461.8 | Ti (II) ($2P_{1/2}$) | 3.0 | 1.8 |
| | | 462.8 | Ti-O ($2P_{1/2}$) | 2.2 | 1.8 |
| | | 464.6 | $TiO_2$ ($2P_{1/2}$) | 18.2 | 2.2 |
| | | 465.9 | Ti-F ($2P_{1/2}$) | 2.8 | 2.2 |
| O 1s | 25.0 | 529.9 | Ti-O | 6.3 | 1.1 |
| | | 532.1 | Ti-OH | 11.0 | 1.5 |
| | | 530.8 | C-Ti-$O_x$ | 53.2 | 1.2 |
| | | 532 | O=C-O | 15.6 | 1.8 |
| | | 533 | C-O | 9.2 | 1.8 |
| | | 532.9 | $H_2O$ | 4.7 | 1.8 |
| C 1s | 32.0 | 281.8 | C-Ti | 6.6 | 0.5 |
| | | 282.5 | C-Ti-O | 0.6 | 0.5 |
| | | 284.9 | C-C | 79.0 | 1.5 |
| | | 286.3 | C-O | 11.0 | 1.5 |
| | | 288.5 | O=C-O | 2.8 | 1.5 |

The 531.9 – 533.1 eV region of the O 1s **(Figure 6e, S22d – S22f** ), consisting of Ti-OH, O-C=O, and C-O component peaks, qualitatively increased as the film pH shifted towards basic. In C 1s (**Figure 6f**, **S22g** – **S22i** and **Table S8** – **S10**), the 4 LP of pH 2.8 multilayer showed the highest C-O, and O-C=O percentage compared to others. These O 1s and C 1s results are consistent with the decrease in No. of MXene layers per LP with increasing pH

that multilayers were deposited at, resulting in the increased amount of PAA probed by SR-XPS beneath the MXenes.

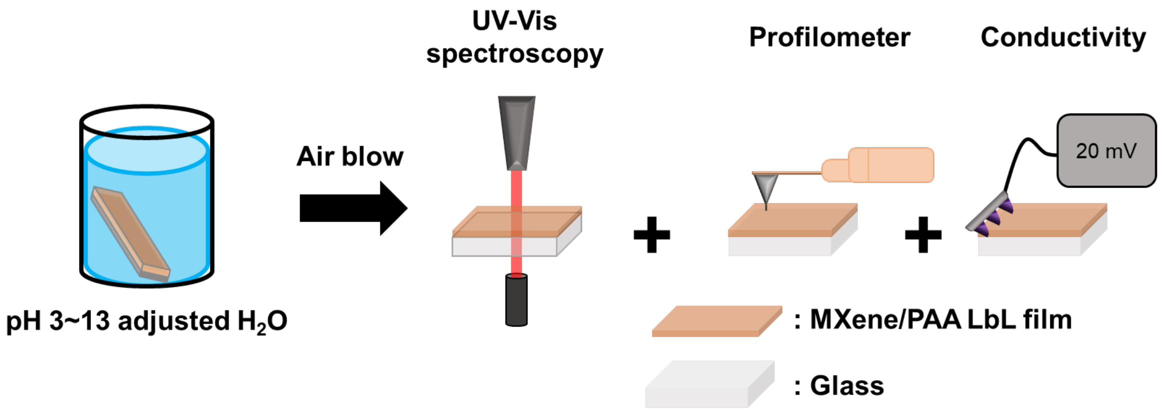


**Figure S23.** Illustration scheme for the experiment of the pH effects on the multilayer stability.

The pH of water was adjusted using 0.1 M of hydrochloric acid and sodium hydroxide and MXene/PAA LbL multilayers were dipped into each pH-adjusted water. Multilayer samples were taken out after a certain period of time and dried by air-blowing and their optical absorbance, film thickness, and conductivity changes were measured.

Conditions

1) The number of LP: 20

2) Aqueous solution pH: 3, 5, 9, 11, 13

3) Time: 0~20 days

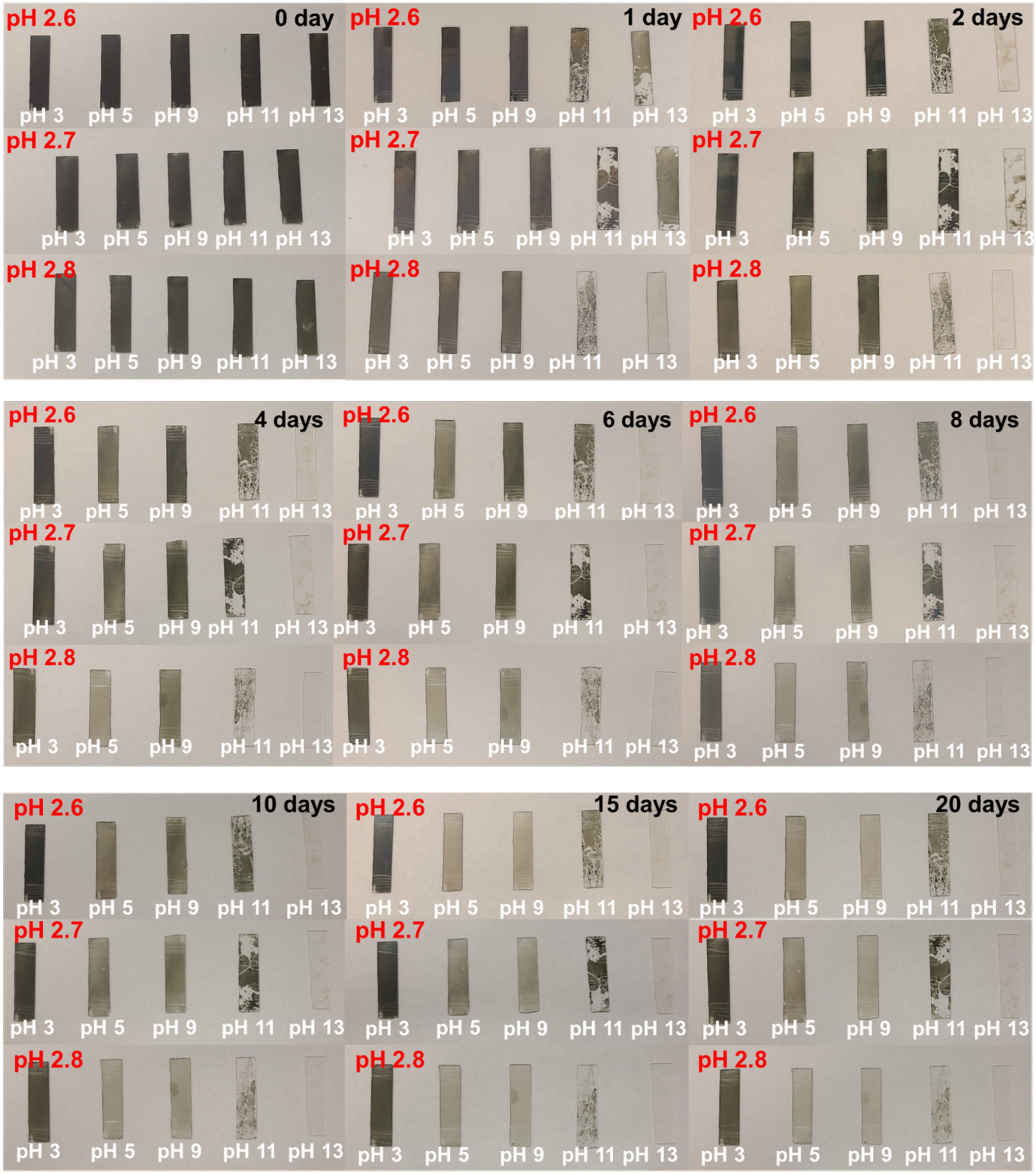


**Figure S24.** Digital images of the changes in MXene/PAA LbL multilayers after being immersed in pH-adjusted aqueous solution for up to 20 days. The red and white letters indicate the film and aqueous pH, respectively.

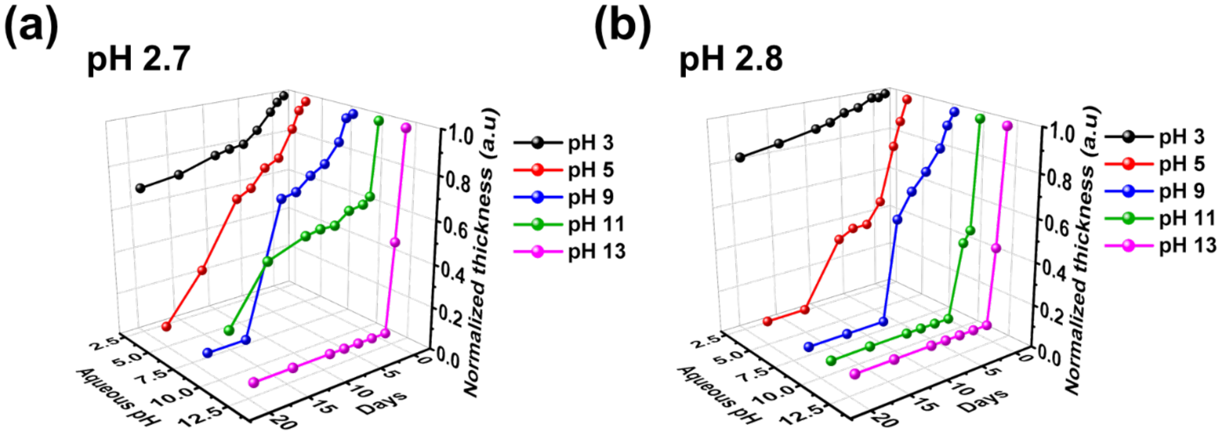


**Figure S25.** Normalized film thickness of (a) pH 2.7, and (b) pH 2.8 as a function of aqueous pH and exposure time.

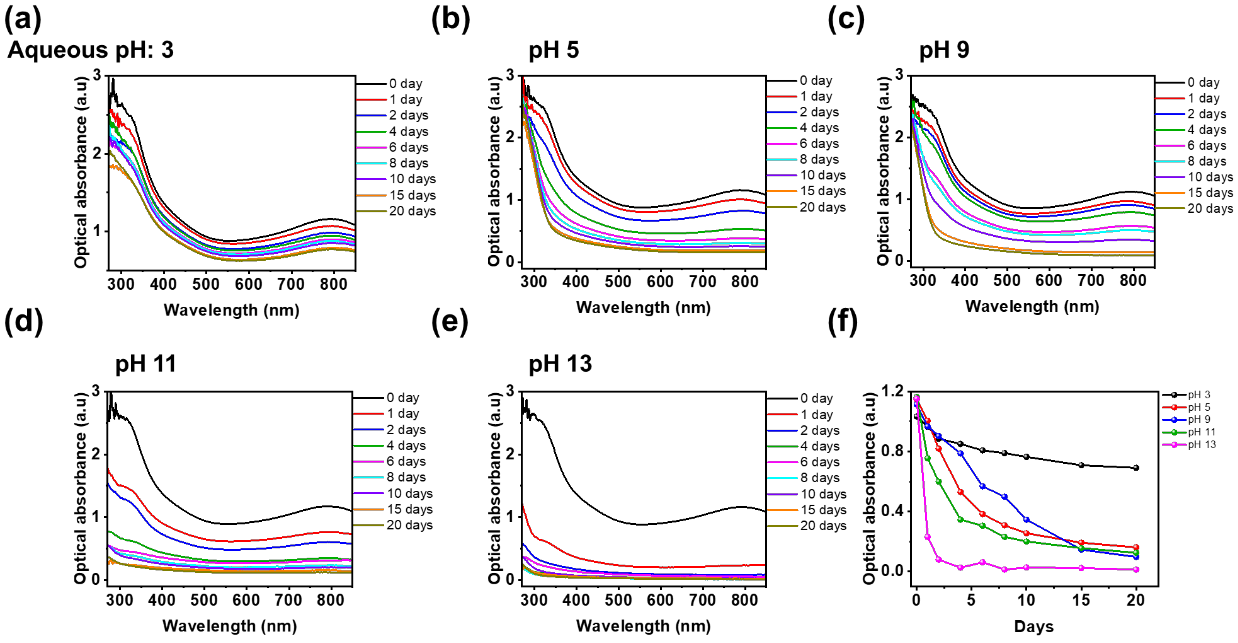

**Figure S26.** Optical absorbance spectrums of pH 2.6 MXene/PAA LbL multilayer after immersion in pH (a) 3, (b) 5, (c) 9, (d) 11, (e) 13 aqueous solutions for up to 20 days. (f) Changes of optical absorbance intensity at 770 nm over time under different pH aqueous solution conditions.

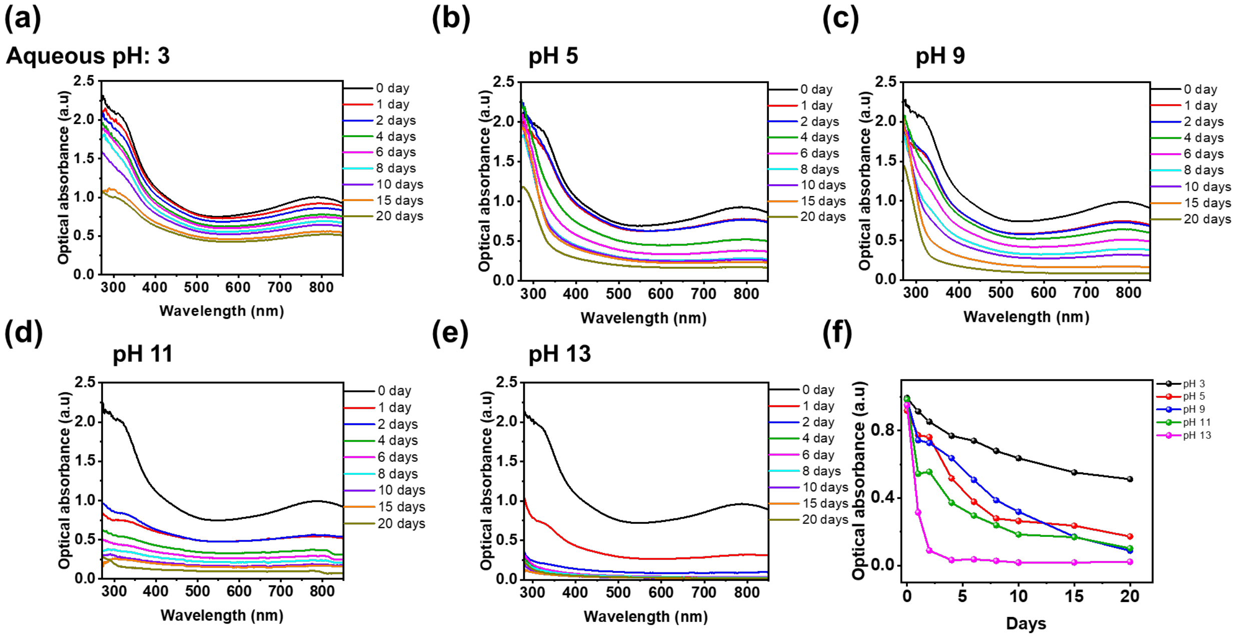


**Figure S27.** Optical absorbance spectrums of pH 2.7 MXene/PAA LbL multilayer after immersion in pH (a) 3, (b) 5, (c) 9, (d) 11, (e) 13 aqueous solutions for up to 20 days. (f) Changes of optical absorbance intensity at 770 nm over time under different pH aqueous solution conditions.

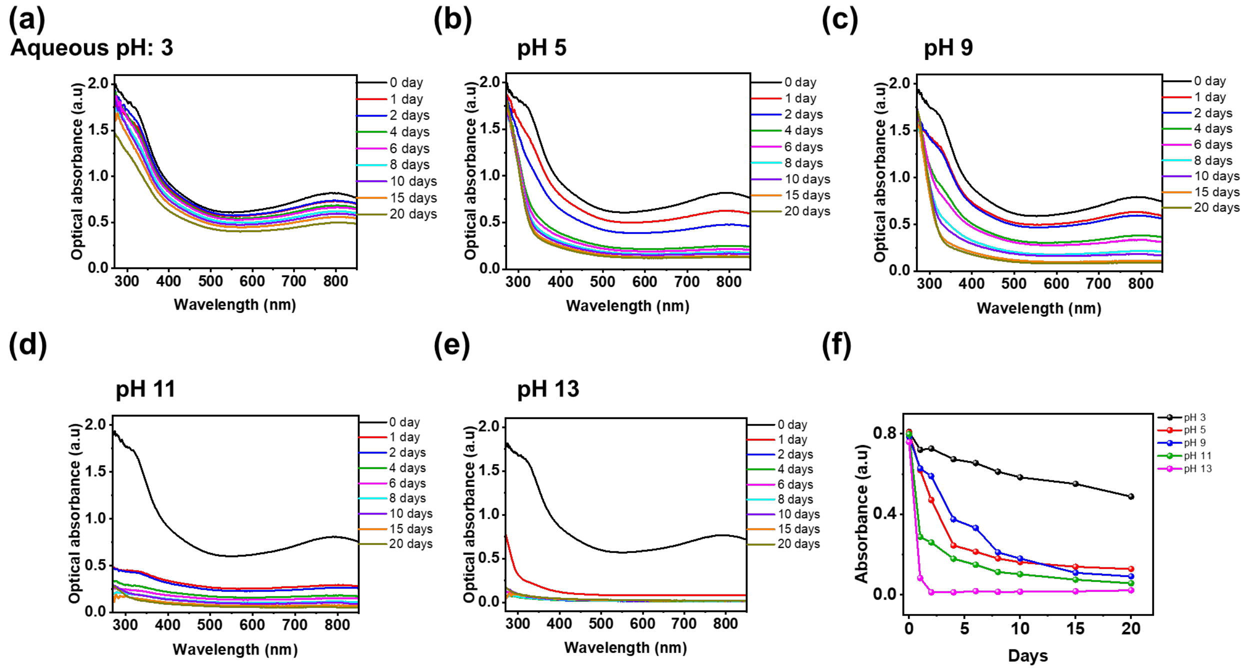


**Figure S28.** Optical absorbance spectrums of pH 2.8 MXene/PAA LbL multilayer after immersion in pH (a) 3, (b) 5, (c) 9, (d) 11, (e) 13 aqueous solutions for up to 20 days. (f) Changes of optical absorbance intensity at 770 nm over time under different pH aqueous solution conditions.

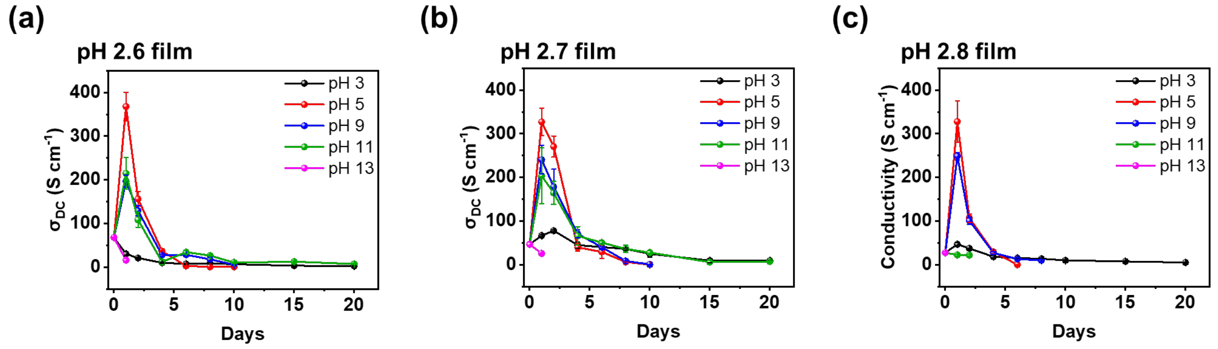


**Figure S29.** $\sigma_{DC}$ changes of (a) pH 2.6, (b) pH 2.7, and (c) pH 2.8 multilayers after immersion in pH 3, 5, 9, 11, and 13 aqueous solutions up to 20 days.

**Table S11.** Comparison of film thickness and $\sigma_{DC}$ for composite films.

| Sample No. | Composite film | Thickness (nm) | $\sigma_{DC}$ (S $cm^{-1}$) | Coating method | Ref. |
|---|---|---|---|---|---|
| 1 | GO | >$10^4$ | 124.2 | Vacuum filtration | 9 |
| 2 | GO-PAA-IV | >×$10^4$ | 108.9 | Vacuum filtration | 9 |
| 3 | Mxene/PVDF | 2.7×$10^4$ | 214.6 | Doctor blading | 10 |
| 4 | MXene/epoxy | 2.0×$10^6$ | 1.1 | evaporation Molding | 11 |
| 5 | MXene/TAEA | 150.0 | 730.0 | LbL | 12 |
| 6 | MXene/Polyurethane | 5.2×$10^4$ | 16.0 | LbLspray coating | 13 |
| 7 | PANI/MXene/CF | 5.5×$10^5$ | 0.2 | Dip coating LbL | 14 |
| 8 | MXene/PDADMA | 37.1 | 37.9 | LbL | 15 |
| 9 | MXene/PDMS | 2.0×$10^4$ | 62.2 | LbL | 16 |
| 10 | $Ti_3C_2T_x$/EP | 2.0×$10^6$ | 452.0 | Mixing | 17 |
| 11 | $Ti_3C_2T_z$/f-$Ti_3C_2T_z$ | 63.0 | 920.0 | LbL | 18 |
| 12 | MX-OE-75 | 6.0×$10^3$ | 29.5 | Vacuum filtration | 19 |
| 13 | MX-OE-80 | 5.3×$10^3$ | 138.2 | Vacuum filtration | 19 |
| 14 | MX-OE-85 | 5.2×$10^3$ | 572.5 | Vacuum filtration | 19 |
| 15 | MX-OE-90 | 5.3×$10^3$ | 1.7×$10^3$ | Vacuum filtration | 19 |
| 16 | $Ti_3C_2$/PVA/MWCNT/PSS | 130.0 | 170.0 | LbL | 20 |
| 17 | pH 2.6 | 145.0 ± 10.2 | 67.8 ± 1.1 | LbL | This work |
| 18 | pH 2.7 | 124.0 ± 9.2 | 46.8 ± 4.9 | LbL | This work |
| 19 | pH 2.8 | 81.7 ± 2.9 | 27.6 ± 1.7 | LbL | This work |
| 20 | pH 2.6_pH 5 | 138.9 ± 5.1 | 368.1 ± 32.6 | LbL | This work |
| 21 | pH 2.6_pH 5 | 102.0 ± 6.1 | 327.0 ± 31.2 | LbL | This work |
| 22 | pH 2.6_pH 5 | 71.7 ± 6.3 | 327.8 ± 47.2 | LbL | This work |

**Equations**

Calculation of PAA weight percentage ($wt_{PAA}$)

$$wt_{PAA}(\%) = 100 \times \frac{\sum m_{PAA}}{\sum m_{PAA} + \sum m_{MXene}} \quad \textbf{Eqn. S1}$$

where $\sum m_{PAA}$ is the sum of the areal mass of each PAA layer (µg cm$^{-2}$), and $\sum m_{MXene}$ is the sum of the MXene layer areal mass (µg cm$^{-2}$).

Calculation of film density ($\rho_{film}$)

$$\rho_{film} = \frac{\sum m_{PAA} + \sum m_{MXene}}{L} \quad \textbf{Eqn. S2}$$

where L is the film thickness coated on the QCM quartz crystal substrate. The No. of MXene layers per LP was calculated using Eqn. 3.

Calculation of $\sigma_{DC}$

$$\sigma_{DC}(S\ cm^{-1}) = \frac{\ln(2) \times I}{\pi \times V \times L} \quad \textbf{Eqn. S3}$$

where I is the current (mA), V is the voltage (mV), and L is the film thickness measured by profilometry (cm).

Calculation of No. of MXene layers per LP

$$\text{No. of MXene layers per LP} = \frac{(\sum m_{MXene})}{\text{No.of MXene layer} \times \rho_{MXene} \times L_{MXene}} \quad \textbf{Eqn. S4}$$

where No. of MXene layer is the number of MXene layers consisting of multilayer (20), $\rho_{MXene}$ is the density of MXene reported in the literature,[21] and $L_{MXene}$ is the thickness of single MXene nanosheet reported in the literature.[21]

**References**

(1) Kumar, S.; Sharma, A.; Tripathi, B.; Srivastava, S.; Agrawal, S.; Singh, M.; Awasthi, K.; Vijay, Y. K. Enhancement of hydrogen gas permeability in electrically aligned MWCNT-PMMA composite membranes. *Micron* **2010**, *41* (7), 909-914. DOI: https://doi.org/10.1016/j.micron.2010.05.016.

(2) Saha, S.; Son, W.; Kim, N. H.; Lee, J. H. Fabrication of impermeable dense architecture containing covalently stitched graphene oxide/boron nitride hybrid nanofiller reinforced semi-interpenetrating network for hydrogen gas barrier applications. *Journal of Materials Chemistry A* **2022**, *10* (8), 4376-4391, 10.1039/D1TA09486F. DOI: 10.1039/D1TA09486F.

(3) Yuan, S.; Sun, Y.; Yang, C.; Zhang, Y.; Cong, C.; Yuan, Y.; Lin, D.; Pei, L.; Zhu, Y.; Wang, H. A novel dual-functional epoxy-based composite coating with exceptional anti-corrosion and enhanced hydrogen gas barrier properties. *Chemical Engineering Journal* **2022**, *449*, 137876. DOI: https://doi.org/10.1016/j.cej.2022.137876.

(4) Li, X.; Bandyopadhyay, P.; Nguyen, T. T.; Park, O.-k.; Lee, J. H. Fabrication of functionalized graphene oxide/maleic anhydride grafted polypropylene composite film with excellent gas barrier and anticorrosion properties. *Journal of Membrane Science* **2018**, *547*, 80-92. DOI: https://doi.org/10.1016/j.memsci.2017.10.031.

(5) Seo, O. B.; Saha, S.; Kim, N. H.; Lee, J. H. Preparation of functionalized MXene-stitched-graphene oxide/poly (ethylene-co-acrylic acid) nanocomposite with enhanced hydrogen gas

barrier properties. *Journal of Membrane Science* **2021**, *640*, 119839. DOI: https://doi.org/10.1016/j.memsci.2021.119839.

(6) Yang, Y.-H.; Bolling, L.; Priolo, M. A.; Grunlan, J. C. Super Gas Barrier and Selectivity of Graphene Oxide-Polymer Multilayer Thin Films. *Advanced Materials* **2013**, *25* (4), 503-508. DOI: https://doi.org/10.1002/adma.201202951.

(7) Rajasekar, R.; Kim, N. H.; Jung, D.; Kuila, T.; Lim, J. K.; Park, M. J.; Lee, J. H. Electrostatically assembled layer-by-layer composites containing graphene oxide for enhanced hydrogen gas barrier application. *Composites Science and Technology* **2013**, *89*, 167-174. DOI: https://doi.org/10.1016/j.compscitech.2013.10.004.

(8) Liu, H.; Bandyopadhyay, P.; Kshetri, T.; Kim, N. H.; Ku, B.-C.; Moon, B.; Lee, J. H. Layer-by-layer assembled polyelectrolyte-decorated graphene multilayer film for hydrogen gas barrier application. *Composites Part B: Engineering* **2017**, *114*, 339-347. DOI: https://doi.org/10.1016/j.compositesb.2017.02.007.

(9) Wan, S.; Hu, H.; Peng, J.; Li, Y.; Fan, Y.; Jiang, L.; Cheng, Q. Nacre-inspired integrated strong and tough reduced graphene oxide–poly(acrylic acid) nanocomposites. *Nanoscale* **2016**, *8* (10), 5649-5656, 10.1039/C6NR00562D. DOI: 10.1039/C6NR00562D.

(10) Li, Y.; Zhou, B.; Shen, Y.; He, C.; Wang, B.; Liu, C.; Feng, Y.; Shen, C. Scalable manufacturing of flexible, durable Ti3C2Tx MXene/Polyvinylidene fluoride film for multifunctional electromagnetic interference shielding and electro/photo-thermal conversion applications. *Composites Part B: Engineering* **2021**, *217*, 108902. DOI: https://doi.org/10.1016/j.compositesb.2021.108902.

(11) Wang, L.; Chen, L.; Song, P.; Liang, C.; Lu, Y.; Qiu, H.; Zhang, Y.; Kong, J.; Gu, J. Fabrication on the annealed Ti3C2Tx MXene/Epoxy nanocomposites for electromagnetic interference

shielding application. *Composites Part B: Engineering* **2019**, *171*, 111-118. DOI: https://doi.org/10.1016/j.compositesb.2019.04.050.

(12) Tian, W.; VahidMohammadi, A.; Wang, Z.; Ouyang, L.; Beidaghi, M.; Hamedi, M. M. Layer-by-layer self-assembly of pillared two-dimensional multilayers. *Nature Communications* **2019**, *10* (1), 2558. DOI: 10.1038/s41467-019-10631-0.

(13) Gao, Q.; Pan, Y.; Zheng, G.; Liu, C.; Shen, C.; Liu, X. Flexible multilayered MXene/thermoplastic polyurethane films with excellent electromagnetic interference shielding, thermal conductivity, and management performances. *Advanced Composites and Hybrid Materials* **2021**, *4* (2), 274-285. DOI: 10.1007/s42114-021-00221-4.

(14) Yin, G.; Wang, Y.; Wang, W.; Yu, D. Multilayer structured PANI/MXene/CF fabric for electromagnetic interference shielding constructed by layer-by-layer strategy. *Colloids and Surfaces A: Physicochemical and Engineering Aspects* **2020**, *601*, 125047. DOI: https://doi.org/10.1016/j.colsurfa.2020.125047.

(15) Echols, I. J.; An, H.; Yun, J.; Sarang, K. T.; Oh, J.-H.; Habib, T.; Zhao, X.; Cao, H.; Holta, D. E.; Radovic, M.; et al. Electronic and Optical Property Control of Polycation/MXene Layer-by-Layer Assemblies with Chemically Diverse MXenes. *Langmuir* **2021**, *37* (38), 11338-11350. DOI: 10.1021/acs.langmuir.1c01904.

(16) Wang, Y.; Li, T.-T.; Shiu, B.-C.; Zhang, X.; Peng, H.-K.; Lou, C.-W.; Lin, J.-H. MXene-coated multi-response conductive film based on layer-by-layer assembly strategy for electromagnetic interference shielding. *Journal of Materials Research and Technology* **2021**, *15*, 6011-6024. DOI: https://doi.org/10.1016/j.jmrt.2021.11.050.

(17) Feng, A.; Hou, T.; Jia, Z.; Zhang, Y.; Zhang, F.; Wu, G. Preparation and Characterization of Epoxy Resin Filled with Ti3C2Tx MXene Nanosheets with Excellent Electric Conductivity. *Nanomaterials* **2020**, *10* (1), 162.

(18) Echols, I. J.; Yun, J.; Cao, H.; Thakur, R. M.; Sarmah, A.; Tan, Z.; Littleton, R.; Radovic, M.; Green, M. J.; Lutkenhaus, J. L. Conformal Layer-by-Layer Assembly of Ti3C2Tz MXene-Only Thin Films for Optoelectronics and Energy Storage. *Chemistry of Materials* **2022**, *34* (11), 4884-4895. DOI: 10.1021/acs.chemmater.1c04394.

(19) Liu, Z.; Zhang, G.; Chen, W.; Wang, J.; Zhang, B.; Zhang, Q. Robust biomimetic Ti3C2Tx nanocomposite films enhanced by mussel-inspired polymer for highly efficient electromagnetic shielding and thermal camouflage. *Carbon* **2022**, *196*, 410-421. DOI: https://doi.org/10.1016/j.carbon.2022.05.004.

(20) Weng, G.-M.; Li, J.; Alhabeb, M.; Karpovich, C.; Wang, H.; Lipton, J.; Maleski, K.; Kong, J.; Shaulsky, E.; Elimelech, M.; et al. Layer-by-Layer Assembly of Cross-Functional Semi-transparent MXene-Carbon Nanotubes Composite Films for Next-Generation Electromagnetic Interference Shielding. *Advanced Functional Materials* **2018**, *28* (44), 1803360. DOI: https://doi.org/10.1002/adfm.201803360.

(21) Zhao, X.; Vashisth, A.; Blivin, J. W.; Tan, Z.; Holta, D. E.; Kotasthane, V.; Shah, S. A.; Habib, T.; Liu, S.; Lutkenhaus, J. L.; et al. pH, Nanosheet Concentration, and Antioxidant Affect the Oxidation of Ti3C2Tx and Ti2CTx MXene Dispersions. *Advanced Materials Interfaces* **2020**, *7* (20), 2000845. DOI: https://doi.org/10.1002/admi.202000845.
